# Supplementary material for: Antibody Fc-receptor FcεR1γ stabilizes cell surface receptors in group 3 innate lymphoid cells and promotes anti-infection immunity
Source: Nat Commun. 2024 Jul 16;15:5981. doi: 10.1038/s41467-024-50266-4 (PMC11252441; doi:10.1038/s41467-024-50266-4)
Supplement: Supplementary file 1 — Supplementary Information [file 41467_2024_50266_MOESM1_ESM.pdf]

Antibody Fc-receptor FcεR1γ stabilizes cell surface receptors in group 3 innate lymphoid cells and promotes anti-infection immunity

Chao Huang[1,2,3,4,#,*], Wenting Zhu[2,3,4,5,#], Qing Li[6,#], Yuchen Lei[1,#], Xi Chen[2,3,4], Shaorui Liu[2,3,4], Dianyu Chen[2,3,4], Lijian Zhong[1], Feng Gao[2,3,4], Shujie Fu[2,3], Danyang He[2,3], Jinsong Li[1,6], Heping Xu[2,3,4,*]

[1]Key Laboratory of Systems Health Science of Zhejiang Province, School of Life Science, Hangzhou Institute for Advanced Study, University of Chinese Academy of Sciences, Hangzhou, China.

[2]Laboratory of Systems Immunology, School of Medicine, Westlake University, Hangzhou, Zhejiang, China.

[3]Key Laboratory of Growth Regulation and Translational Research of Zhejiang Province, School of Life Sciences, Westlake University, Hangzhou, Zhejiang, China.

[4]Center for Infectious Disease Research, Westlake Laboratory of Life Sciences and Biomedicine, Hangzhou, Zhejiang, China.

[5]School of Pharmaceutical Science and Technology, Hangzhou Institute for Advanced Study, University of Chinese Academy of Sciences, Hangzhou, China.

[6]Key Laboratory of Multi-Cell Systems, Shanghai Key Laboratory of Molecular Andrology, CAS Center for Excellence in Molecular Cell Science, Shanghai Institute of Biochemistry and Cell Biology, Chinese Academy of Sciences, University of Chinese Academy of Sciences, Shanghai, China.

[#] Equally Contributing Authors.

* Corresponding Authors: huangchao@ucas.ac.cn (C. H.), xuheping@westlake.edu.cn (H.X.)

Figure S1 page 1

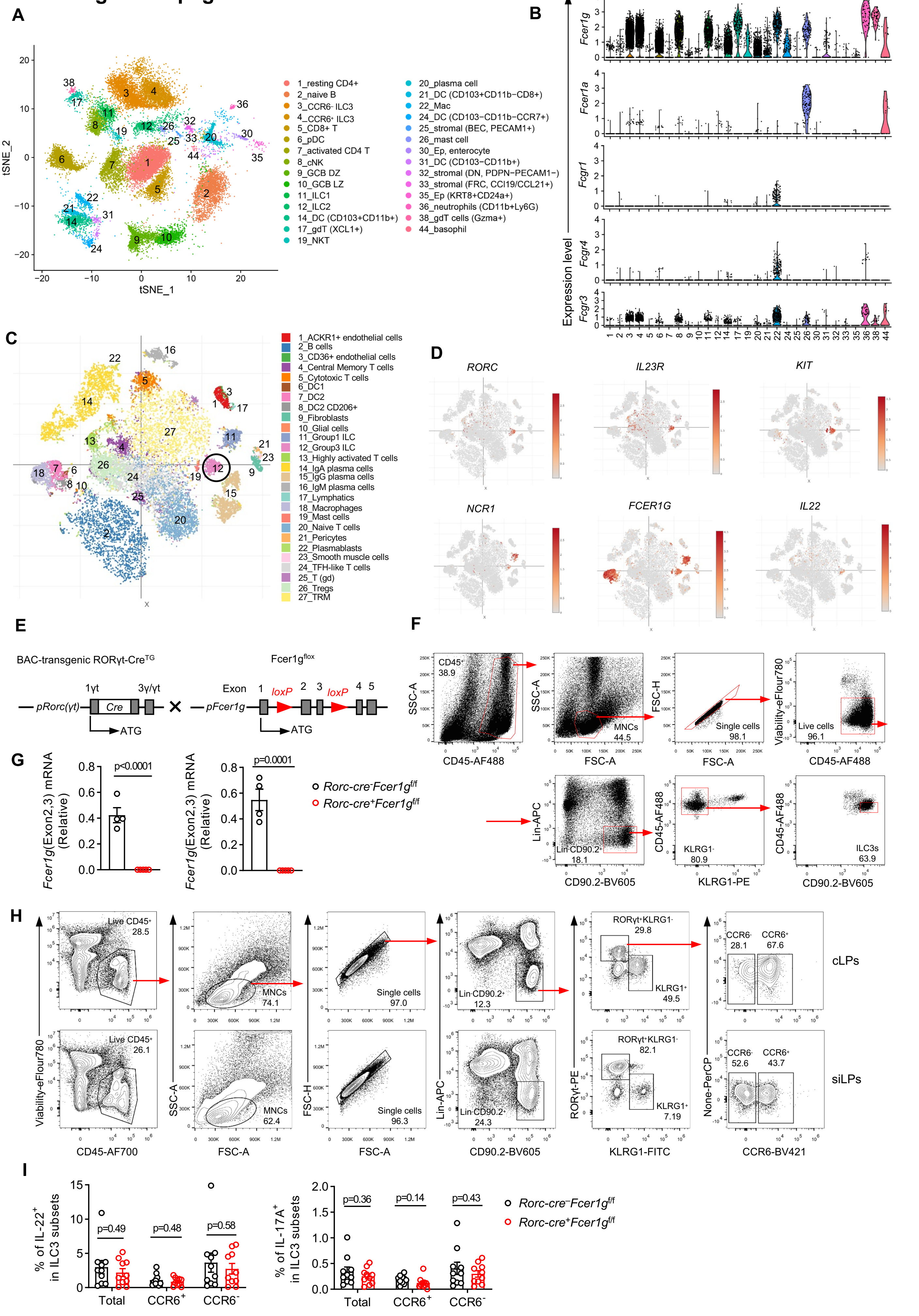

Figure S1 page 2

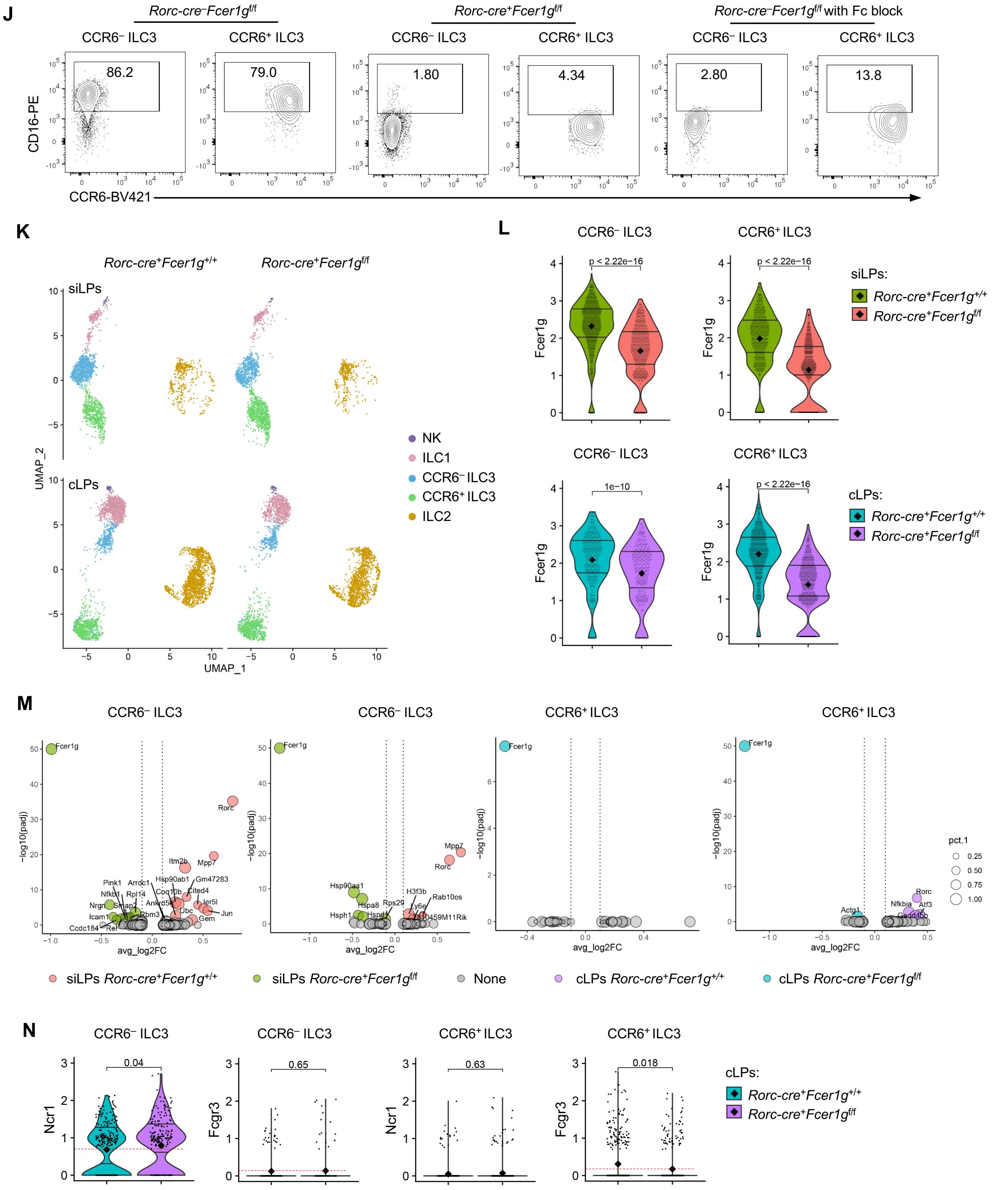

Supplementary Figure 1. Human and murine ILC3s highly express *Fcer1g*. Related to Fig. 1

A T-distributed Stochastic Neighbourhood Embedding (tSNE) plot showing cell clusters of published scRNA-seq dataset of intestinal $CD45^+$ cells in WT mice (GEO: GSE124880)

B Violin plots of distribution of mean expression levels (log_2(TPM+1), y axis) of genes encoding antibody Fc receptors in clusters from (**A**).

C Cell clusters of the human intestinal immune cells using droplet-based scRNA-seq. The data are derived from human uninflamed ileum. (https://singlecell.broadinstitute.org/single_cell/study/SCP359).

D Relative expressions of *RORC*, *IL-23R*, *KIT*, *NCR1, FCER1G, IL22,* are colored by two-dimensional embedding as in (**C**).

E Diagram for generation of *Rorc-cre Fcer1g*^{flox} mice.

F, G Gating strategy for sorting total ILC3s from small intestinal LPs (siLPs) (**F**). Quantification of exon2-3 of *Fcer1g* transcripts in ILC3s (**G**, sorted as in **F**) of siLPs (left) and colonic LPs (cLPs, right) from *Rorc-cre*$^-$*Fcer1g*$^{f/f}$ (*n*=4 mice) mice and *Rorc-cre*$^+$*Fcer1g*$^{f/f}$ (*n*=5 mice) mice. Lin: Lineage markers.

H Gating strategy for flow cytometry analysis of ILC3s from cLPs (top) and siLPs (bottom).

I Quantification of the frequencies of IL-22- and IL-17A-expressing cells in total, $CCR6^+$ and $CCR6^-$ ILC3s (as gated in **Fig. S2C**) from siLPs of indicated mice at steady state (**Methods**). *n*=10 mice per group.

J Representative flow plots showing the expression of CD16 in $CCR6^+$ and $CCR6^-$ ILC3 subsets from siLPs of mice with the indicated genotype. Fc block represents co-incubation with purified anti-mouse CD16/CD32 (Clone: 2.4G2) antibody.

K-N Cell clusters from **Fig. 1H** split by tissues and genotypes (**K**). Violin plots showing expression levels of *Fcer1g* (**L**), *Fcgr3* and *Ncr1* (**N**) in clusters $CCR6^-$ ILC3 and $CCR6^+$ ILC3 from **Fig. 1H**. Volcano plots showing differentially expressed genes (DEGs) of scRNA-seq profiles from **Fig. 1H** (**M**).

Data are pooled from two (**F, G, I**) or three (**H**) independent experiments shown as the mean ± SEM. Statistical significance was tested by two-tailed *t* test (**G, I**) or two-tailed wilcoxon test (**L, N**).

Figure S2

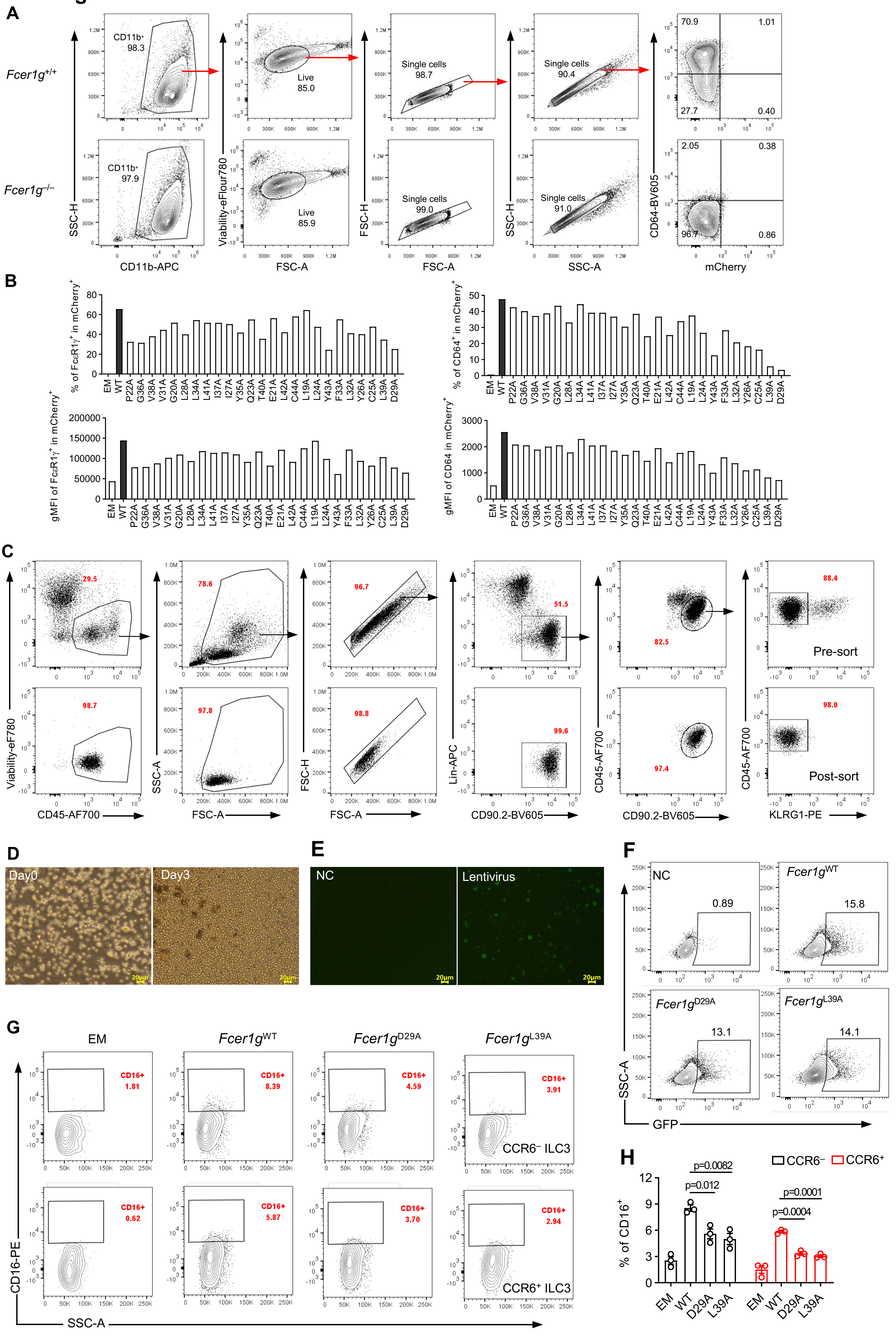
A
Fcer1g+/+
Fcer1g−/−
CD11b+ 98.3
CD11b+ 97.9
Live 85.0
Live 85.9
Single cells 98.7
Single cells 99.0
Single cells 90.4
Single cells 91.0
70.9
1.01
27.7
0.40
2.05
0.38
96.7
0.86
SSC-H
Viability-eFlour780
FSC-H
SSC-H
CD64-BV605
CD11b-APC
FSC-A
FSC-A
SSC-A
mCherry
B
% of FcεR1γ+ in mCherry+
% of CD64+ in mCherry+
gMFI of FcεR1γ+ in mCherry+
gMFI of CD64 in mCherry+
EM WT P22A G36A V38A V31A G20A L28A L34A L41A I37A I27A Y35A Q23A T40A E21A L42A C44A L19A L24A Y43A F33A L32A Y26A C25A L39A D29A
C
29.5
78.6
96.7
51.5
82.5
88.4
98.7
97.8
98.8
99.6
97.4
98.0
Pre-sort
Post-sort
Viability-eF780
SSC-A
FSC-H
Lin-APC
CD45-AF700
CD45-AF700
CD45-AF700
FSC-A
FSC-A
CD90.2-BV605
CD90.2-BV605
KLRG1-PE
D
Day0
Day3
E
NC
Lentivirus
F
NC 0.89
Fcer1gWT 15.8
Fcer1gD29A 13.1
Fcer1gL39A 14.1
SSC-A
GFP
G
EM
Fcer1gWT
Fcer1gD29A
Fcer1gL39A
CD16+ 1.81
CD16+ 8.39
CD16+ 4.59
CD16+ 3.91
CCR6− ILC3
CD16+ 0.62
CD16+ 5.87
CD16+ 3.70
CD16+ 2.94
CCR6+ ILC3
CD16-PE
SSC-A
H
CCR6−
CCR6+
p=0.0082
p=0.012
p=0.0001
p=0.0004
% of CD16+
EM WT D29A L39A EM WT D29A L39A

Supplementary Figure 2. Alanine scanning mutagenesis of FcεR1γ for interaction with CD64 and the validation in ILC3s. Related to Fig. 2

A Representative flow plots showing the expression of CD64 on *Fcer1g* KO or WT BMDMs on day 3.5 post induction as shown in Fig. 2A.

B The percentages and MFIs of FcεR1γ or CD64 on infected cells (mCherry^{+}) were determined by FACS, respectively.

C Gating strategy for sorting ILC3s from the siLPs of *Rag1*$^{-/-}$ *Rorc-cre*$^{+/-}$ *Fcer1g*$^{f/f}$ mice and showing the purity of sorted cells.

D-F Representative images showing ILC3 expansion (**D**) and GFP expression at 48 hours after lentivirus infection analyzed by fluorescence microscope (**E**) or flow cytometry (**F**). NC: uninfected ILC3s as negative control.

G,H Representative flow plots showing the expression of CD16 (**G**) and the quantification of CD16^{+} (**H**, n=3 replicates per group) in ILC3s that express WT or mutated FcεR1γ as indicated, GFP^{+} cells were shown.

Data are representative of two (**A-G**) or are pooled from two (**H**) independent experiments shown as the mean ± SEM. Statistical significance was tested by two-tailed *t* test (**H**).

Figure S3

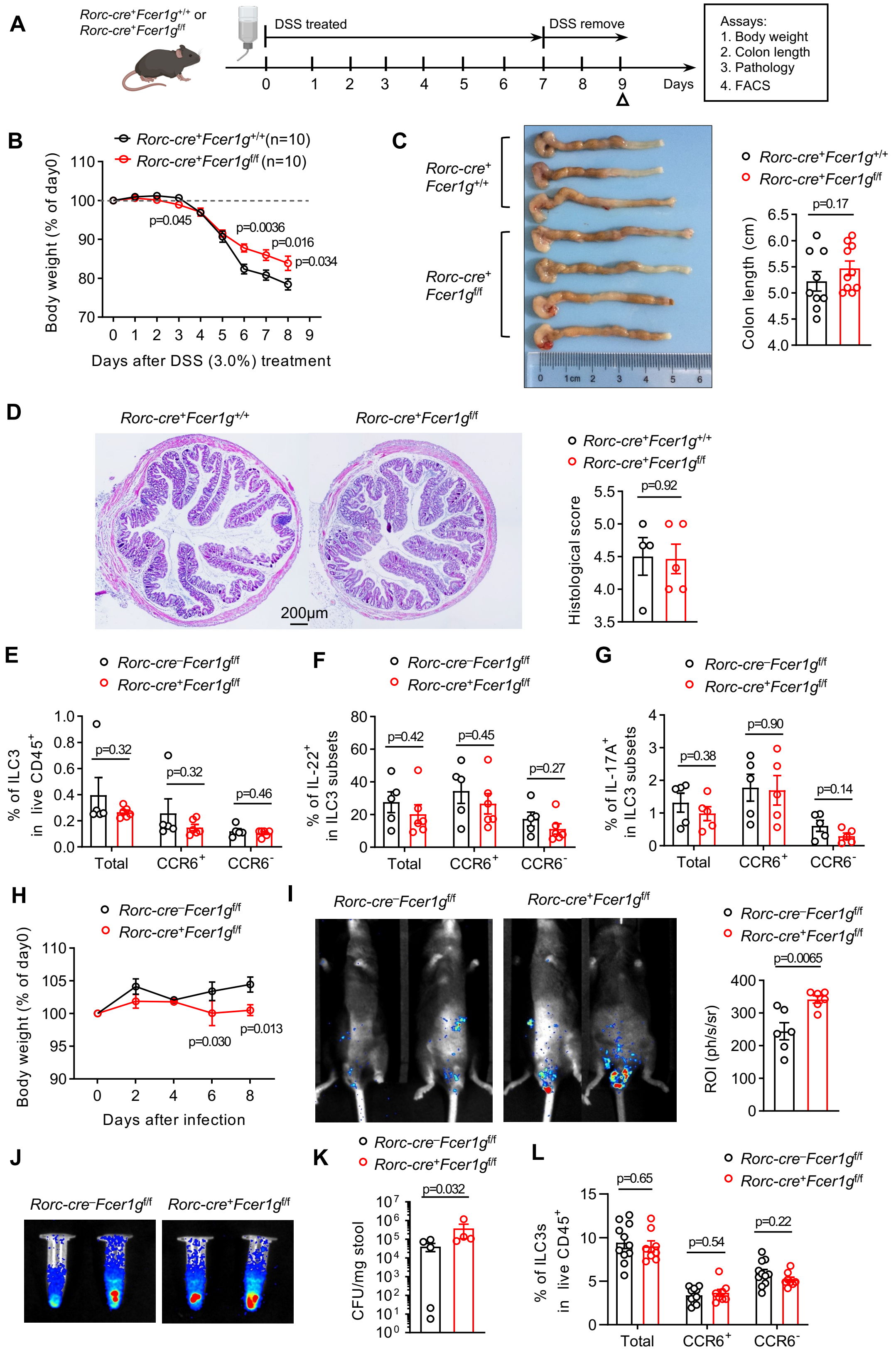

A
Rorc-cre+Fcer1g+/+ or
Rorc-cre+Fcer1gf/f
DSS treated
DSS remove
0
1
2
3
4
5
6
7
8
9
Days
Assays:
1. Body weight
2. Colon length
3. Pathology
4. FACS
B
Rorc-cre+Fcer1g+/+(n=10)
Rorc-cre+Fcer1gf/f (n=10)
Body weight (% of day0)
p=0.045
p=0.0036
p=0.016
p=0.034
Days after DSS (3.0%) treatment
C
Rorc-cre+ Fcer1g+/+
Rorc-cre+ Fcer1gf/f
Rorc-cre+Fcer1g+/+
Rorc-cre+Fcer1gf/f
p=0.17
Colon length (cm)
D
Rorc-cre+Fcer1g+/+
Rorc-cre+Fcer1gf/f
200μm
p=0.92
Histological score
E
Rorc-cre−Fcer1gf/f
Rorc-cre+Fcer1gf/f
% of ILC3 in live CD45+
p=0.32
p=0.32
p=0.46
Total
CCR6+
CCR6-
F
% of IL-22+ in ILC3 subsets
p=0.42
p=0.45
p=0.27
G
% of IL-17A+ in ILC3 subsets
p=0.38
p=0.90
p=0.14
H
Body weight (% of day0)
p=0.030
p=0.013
Days after infection
I
Rorc-cre−Fcer1gf/f
Rorc-cre+Fcer1gf/f
p=0.0065
ROI (ph/s/sr)
J
K
p=0.032
CFU/mg stool
L
p=0.65
p=0.54
p=0.22
% of ILC3s in live CD45+

Supplementary Figure 3. FcεR1γ expression in ILC3s promotes the inflammation induced by *C. rodentium* infection but not DSS. Related to Fig. 3

A-G Experimental scheme of DSS-induced acute colitis (**A**, **Methods**, created with BioRender.com). Body weight change during the treatment (**B**, *n*=10 mice per group). Colon length of mice from both groups on day 9 post initial treatment (**C**, *Rorc-cre*$^{+}$*Fcer1g*$^{+/+}$, *n*=9 mice; *Rorc-cre*$^{+}$*Fcer1g*$^{f/f}$, *n*=10 mice). Representative images of HE staining (left) were shown and histological scores (right) were calculated (**D**, *Rorc-cre*$^{+}$*Fcer1g*$^{+/+}$, *n*=4 mice; *Rorc-cre*$^{+}$*Fcer1g*$^{f/f}$, *n*=5 mice). Quantification of total, CCR6^{+} and CCR6^{-} ILC3s in cLPs of *Rorc-cre*$^{-}$*Fcer1g*$^{f/f}$ (*n*=5) and *Rorc-cre*$^{+}$*Fcer1g*$^{f/f}$ (*n*=6) mice on day 9 post initial treatment (**E**). Quantification of the frequencies of IL-22 (**F**) or IL-17A (**G**) -expressing cells in each compartment from cLPs (*Rorc-cre*$^{-}$*Fcer1g*$^{f/f}$, *n*=5 mice; *Rorc-cre*$^{+}$*Fcer1g*$^{f/f}$, *n*=6 mice). The cells were stimulated with Cell Stimulation Cocktail, Protein Transport Inhibitor Cocktail and rmIL-23 in complete RPMI medium for 2.5 hours before staining (**Methods**).

H-L Mice were infected with *C. rodentium* as in Fig. 3A. Body weight changes were recorded every two days (**H**, *n*=3 mice per group). Representative images of bacterial load determined by bioluminescent imaging in the live animals and the quantification (**I**, *n*=6 mice per group). Representative images of bacterial load determined by bioluminescent imaging in the freshly collected stool (**J**). Viable counts of *C. rodentium* (given as CFU per milligram of stool) determined by culture in the plates (**K**, *Rorc-cre*$^{-}$*Fcer1g*$^{f/f}$, *n*=5 mice; *Rorc-cre*$^{+}$*Fcer1g*$^{f/f}$, *n*=4 mice). The frequencies of total, CCR6^{+} and CCR6^{-} ILC3s from Fig.3F analyzed by surface marker staining (**L**, *Rorc-cre*$^{-}$*Fcer1g*$^{f/f}$, *n*=11 mice; *Rorc-cre*$^{+}$*Fcer1g*$^{f/f}$, *n*=8 mice).

Data are representative of three (**A-D**) or two (**E-K**) or are pooled from two (**L**) independent experiments shown as the mean ± SEM. Statistical significance was tested by two-tailed *t* test (**B, C, D, H, I, L**) or Mann-Whitney U-test (**E, F, G, K**).

Figure S4

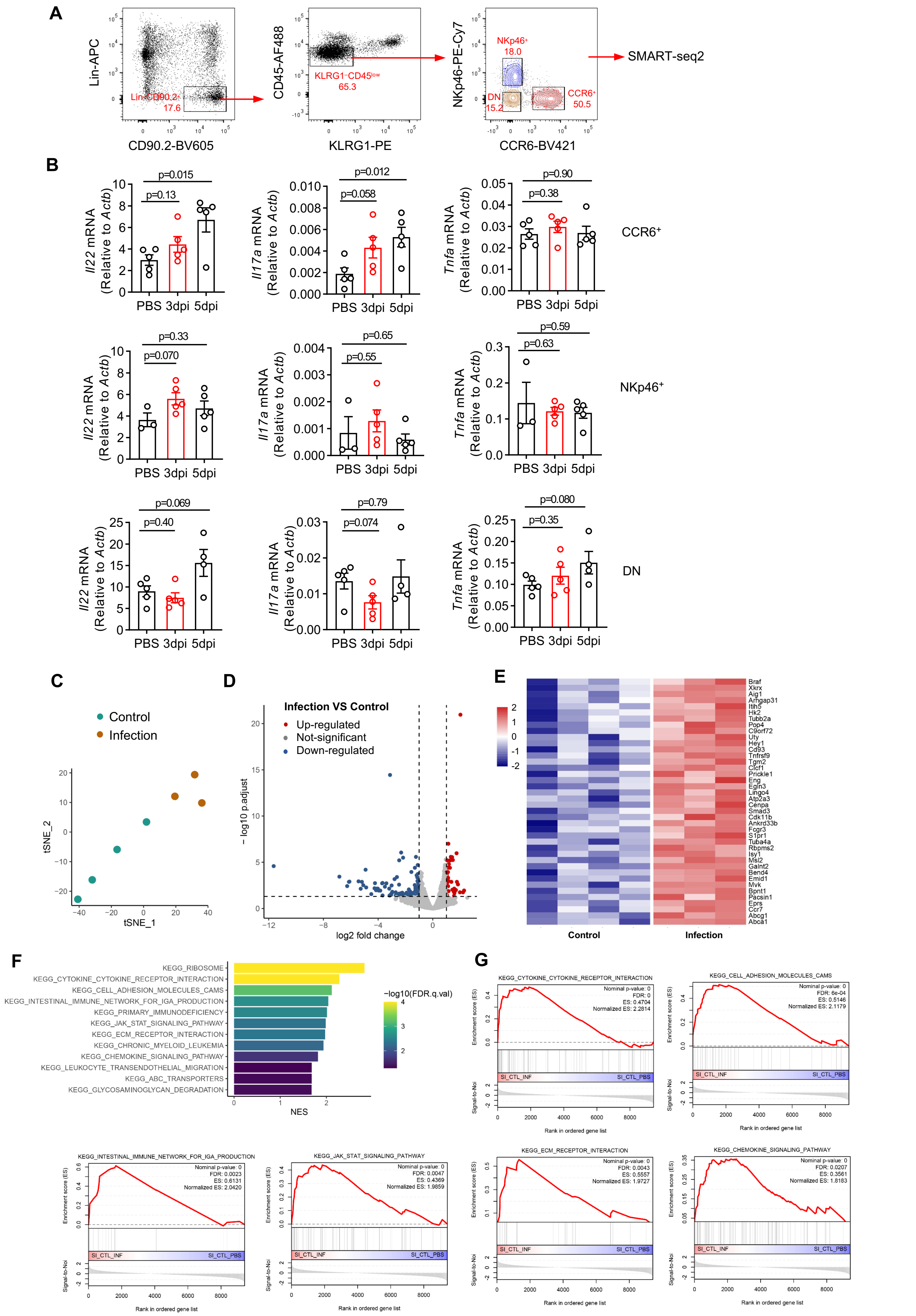
A
Lin-APC
CD90.2-BV605
Lin-CD90.2+
17.6
CD45-AF488
KLRG1-PE
KLRG1-CD45low
65.3
NKp46-PE-Cy7
CCR6-BV421
NKp46+
18.0
DN
15.2
CCR6+
50.5
SMART-seq2
B
Il22 mRNA (Relative to Actb)
Il17a mRNA (Relative to Actb)
Tnfa mRNA (Relative to Actb)
PBS 3dpi 5dpi
p=0.015
p=0.13
p=0.012
p=0.058
p=0.90
p=0.38
CCR6+
p=0.33
p=0.070
p=0.65
p=0.55
p=0.59
p=0.63
NKp46+
p=0.069
p=0.40
p=0.79
p=0.074
p=0.080
p=0.35
DN
C
Control
Infection
tSNE_1
tSNE_2
D
Infection VS Control
Up-regulated
Not-significant
Down-regulated
log2 fold change
– log10 p.adjust
E
Braf
Xkrx
Aig1
Arhgap31
Itih5
Hk2
Tubb2a
Pop4
C9orf72
Uty
Hey1
Cd93
Tnfrsf9
Tgm2
Clcf1
Prickle1
Eng
Egln3
Lingo4
Atp2a3
Cenpa
Smad3
Cdk11b
Ankrd33b
Fcgr3
S1pr1
Tuba4a
Rbpms2
Isy1
Msl2
Galnt2
Bend4
Emid1
Mvk
Bpnt1
Pacsin1
Eprs
Ccr7
Abcg1
Abca1
Control
Infection
F
KEGG_RIBOSOME
KEGG_CYTOKINE_CYTOKINE_RECEPTOR_INTERACTION
KEGG_CELL_ADHESION_MOLECULES_CAMS
KEGG_INTESTINAL_IMMUNE_NETWORK_FOR_IGA_PRODUCTION
KEGG_PRIMARY_IMMUNODEFICIENCY
KEGG_JAK_STAT_SIGNALING_PATHWAY
KEGG_ECM_RECEPTOR_INTERACTION
KEGG_CHRONIC_MYELOID_LEUKEMIA
KEGG_CHEMOKINE_SIGNALING_PATHWAY
KEGG_LEUKOCYTE_TRANSENDOTHELIAL_MIGRATION
KEGG_ABC_TRANSPORTERS
KEGG_GLYCOSAMINOGLYCAN_DEGRADATION
NES
−log10(FDR.q.val)
G
KEGG_CYTOKINE_CYTOKINE_RECEPTOR_INTERACTION
Nominal p-value: 0
FDR: 0
ES: 0.4704
Normalized ES: 2.2814
KEGG_CELL_ADHESION_MOLECULES_CAMS
Nominal p-value: 0
FDR: 6e-04
ES: 0.5146
Normalized ES: 2.1179
KEGG_INTESTINAL_IMMUNE_NETWORK_FOR_IGA_PRODUCTION
Nominal p-value: 0
FDR: 0.0023
ES: 0.6131
Normalized ES: 2.0420
KEGG_JAK_STAT_SIGNALING_PATHWAY
Nominal p-value: 0
FDR: 0.0047
ES: 0.4369
Normalized ES: 1.9859
KEGG_ECM_RECEPTOR_INTERACTION
Nominal p-value: 0
FDR: 0.0043
ES: 0.5557
Normalized ES: 1.9727
KEGG_CHEMOKINE_SIGNALING_PATHWAY
Nominal p-value: 0
FDR: 0.0207
ES: 0.3561
Normalized ES: 1.8183
Enrichment score (ES)
Signal-to-Noi
SI_CTL_INF
SI_CTL_PBS
Rank in ordered gene list

Supplementary Figure 4. Intestinal ILC3s respond to invasive fungal infection. Related to Fig. 4

A Representative flow plots showing the sorting strategy (gated on $CD45^+$ live single cells) for $CCR6^+$, $NKp46^+$, DN ILC3s from siLPs of *Rorc-cre⁻Fcer1g*$^{f/f}$ mice on day 3 and 5 post *C. albicans* infection as in **Fig. 4A**. DN represents $CCR6^-NKp46^-$.

B Quantification of the abundance of *Il22*, *Il17a*, and *Tnfα* transcripts in ILC3s sorted from (**A**) ($CCR6^+$ ILC3s: *n*=5 mice per group. $NKp46^+$ ILC3s: PBS, *n*=3 mice; 3dpi (day post infection), *n*=5 mice; 5dpi, *n*=5 mice. DN ILC3s: PBS, *n*=5 mice; 3dpi, *n*=5 mice; 5dpi, *n*=4 mice).

C-E Gene expression libraries of $CCR6^+$ ILC3s on day 5 post the infection from (**A**) were constructed by SMART-seq2. tSNE plot showing gene expression matrix (**C**). The volcano map showing the DEGs calculated by edgeR (**D**). p.adjust<0.05; fold change>2. Heatmap (**E**) showing the expression (color bar, Z score) of genes (rows) significantly induced in ILC3s by the fungal infection across different conditions (columns). p.adjust< 0.05; fold change >2. (Control, *n*=4 mice; infection, *n*=3 mice).

F-G Gene Set Enrichment Analysis (GSEA) of (**C**). Bar plot showing the top enriched pathways in the infected group (**F**). GSEA enrichment plot showing the detailed results of the pathways enriched in the infected group (**G**).

Data are from two (**A-G**) independent experiments shown as the mean ± SEM. Statistical significance was tested by two-tailed *t* test (**B**).

Figure S5

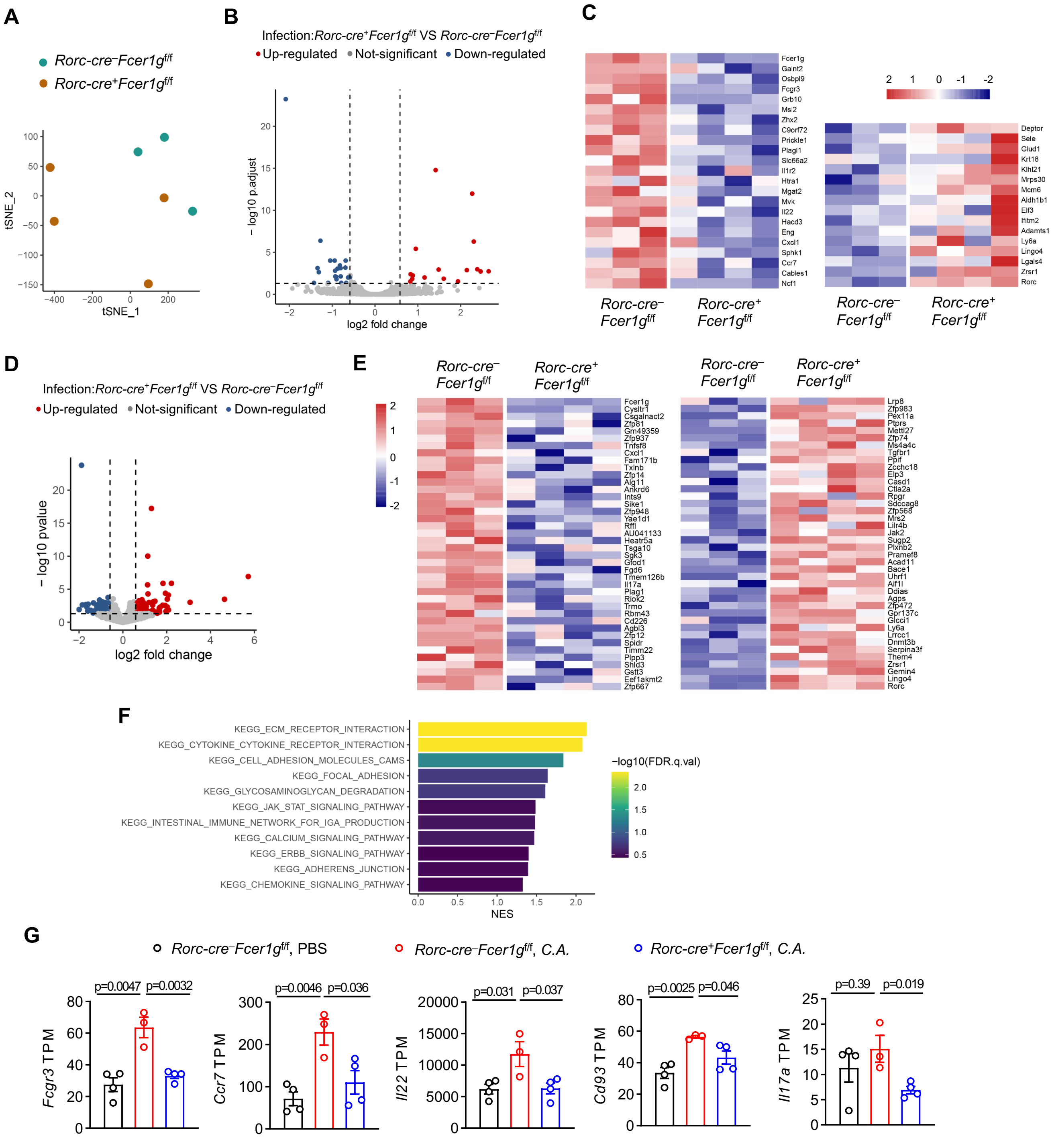

Supplementary Figure 5. FcεR1γ expression in ILC3s maintains transcriptional states in ILC3s in response to fungal infection. Related to Fig. 5

A-C tSNE plot showing the gene expression matrix of CCR6$^+$ ILC3s from siLPs of *Rorc-cre⁻Fcer1g*$^{f/f}$ mice (*n*=3) and *Rorc-cre*$^+$*Fcer1g*$^{f/f}$ mice (*n*=4) on day 5 post *C. albicans* (*C.A.*) infection (**A**). The volcano map (**B**) showing the DEGs of two groups from (**A**), calculated by edgeR. p.adjust<0.05; fold change >1.5. Heatmap (**C**) showing the expression (color bar, Z score) of DEGs (rows) from (**B**) of different groups (columns).

D, E The volcano map (**D**) showing the DEGs of CCR6$^+$ ILC3s isolated and sorted from cLPs of *Rorc-cre⁻Fcer1g*$^{f/f}$ mice (*n*=3) and *Rorc-cre*$^+$*Fcer1g*$^{f/f}$ mice (*n*=4) on day 5 post *C. albicans* infection, calculated by edgeR. p-value< 0.05; fold change>1.5. Heatmap (**E**) showing the expression (color bar, Z score) of top 40 DEGs (rows) from (**D**) of different groups (columns).

F Bar plot showing the negatively enriched pathways in the CKO ILC3s versus control ILC3s in GSEA for (**A**).

G The expression levels (TPM) of *Fcgr3, Ccr7, Il22, Cd93, Il17a* in bulk RNA-seq in all groups (*Rorc-cre⁻Fcer1g*$^{f/f}$ PBS, *n*=4 mice; *Rorc-cre⁻Fcer1g*$^{f/f}$ *C.A.*, *n*=3 mice; *Rorc-cre*$^+$*Fcer1g*$^{f/f}$ *C.A.*, *n*=4 mice).

Data are from two (**A-G**) independent experiments shown as the mean ± SEM. Statistical significance was tested by two-tailed *t* test (**G**).

Figure S6

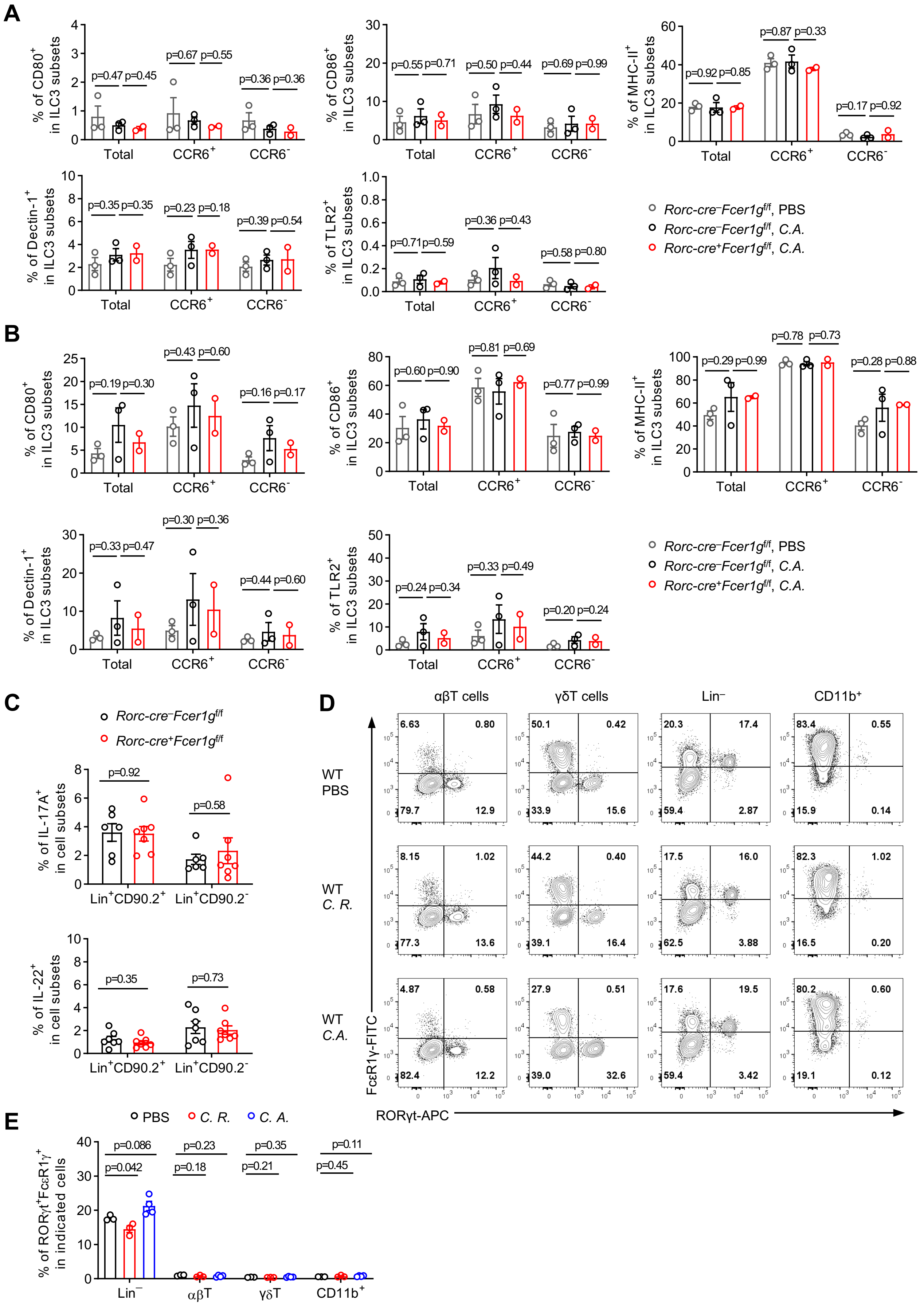

A
% of CD80+ in ILC3 subsets
% of CD86+ in ILC3 subsets
% of MHC-II+ in ILC3 subsets
% of Dectin-1+ in ILC3 subsets
% of TLR2+ in ILC3 subsets
Total
CCR6+
CCR6-
Rorc-cre−Fcer1gf/f, PBS
Rorc-cre−Fcer1gf/f, C.A.
Rorc-cre+Fcer1gf/f, C.A.
B
C
Rorc-cre−Fcer1gf/f
Rorc-cre+Fcer1gf/f
% of IL-17A+ in cell subsets
% of IL-22+ in cell subsets
Lin+CD90.2+
Lin+CD90.2-
D
αβT cells
γδT cells
Lin−
CD11b+
WT PBS
WT C. R.
WT C.A.
FcεR1γ-FITC
RORγt-APC
E
PBS
C. R.
C. A.
% of RORγt+FcεR1γ+ in indicated cells
Lin−
αβT
γδT
CD11b+

Supplementary Figure 6. Expression of *C. albicans*-sensing receptors. Related to Fig. 5 and Fig. 6

A-B The expression levels of CD80, CD86, MHC-II, Dectin-1, TLR2 on total, CCR6^{+} and CCR6^{-} ILC3s from siLPs (**A**) and mesenteric lymph nodes (**B**) of *Rorc*-cre^{-}*Fcer1g*$^{f/f}$ and *Rorc*-cre^{+} *Fcer1g*$^{f/f}$ mice on day 5 post *C. albicans* (*C.A.*) infection, determined by FACS. *Rorc*-cre^{-}*Fcer1g*$^{f/f}$ treated with vehicle (PBS) were as control (*Rorc-cre*$^{-}$*Fcer1g*$^{f/f}$ PBS, *n*=3 mice; *Rorc-cre*$^{-}$*Fcer1g*$^{f/f}$ *C.A.*, *n*=3 mice; *Rorc-cre*$^{+}$*Fcer1g*$^{f/f}$ *C.A.*, *n*=2 mice).

C Quantification of the frequencies of IL-17A and IL-22 expressing cells in each Lin^{+}CD90.2^{+} or Lin^{+} CD90.2^{-} cells from siLPs of *Rorc*-cre^{-}*Fcer1g*$^{f/f}$ and *Rorc*-cre^{+} *Fcer1g*$^{f/f}$ mice on day 5 post *C. albicans* infection. The cells were stimulated with Cell Stimulation Cocktail, Protein Transport Inhibitor Cocktail and rmIL-23 in complete RPMI medium for 4 hours before staining (**Methods**) (For IL-17A: *Rorc-cre*$^{-}$*Fcer1g*$^{f/f}$, *n*=6 mice; *Rorc-cre*$^{+}$*Fcer1g*$^{f/f}$, *n*=7 mice. For IL-22: *n*=7 mice per group).

D-E Representative flow plots (**D**) showing co-expression of FcεR1γ and RORγt in Lin^{-} cells (CD45^{+}CD19^{-}CD3^{-}CD11b^{-}), αβT cells (CD45^{+}CD3^{+}γδTCR^{-}), γδT cells (CD45^{+}CD3^{+}γδTCR^{+}) and CD11b^{+} cells (CD45^{+}CD11b^{+}) in siLPs from mice on day 5 post *C. rodentium* (*C.R.*) or *C.A.* infection. Quantification of the frequencies of FcεR1γ^{+}RORγt^{+} cells (**E**) from (**D**) (PBS, *n*=3 mice; *C.R.*, *n*=3 mice; *C.A.*, *n*=4 mice).

Data are representative of three (**C**) or one (**A, B, D, E**) independent experiments shown as the mean ± SEM.
Statistical significance was tested by two-tailed *t* test (**A, B, C, E**).

Figure S7

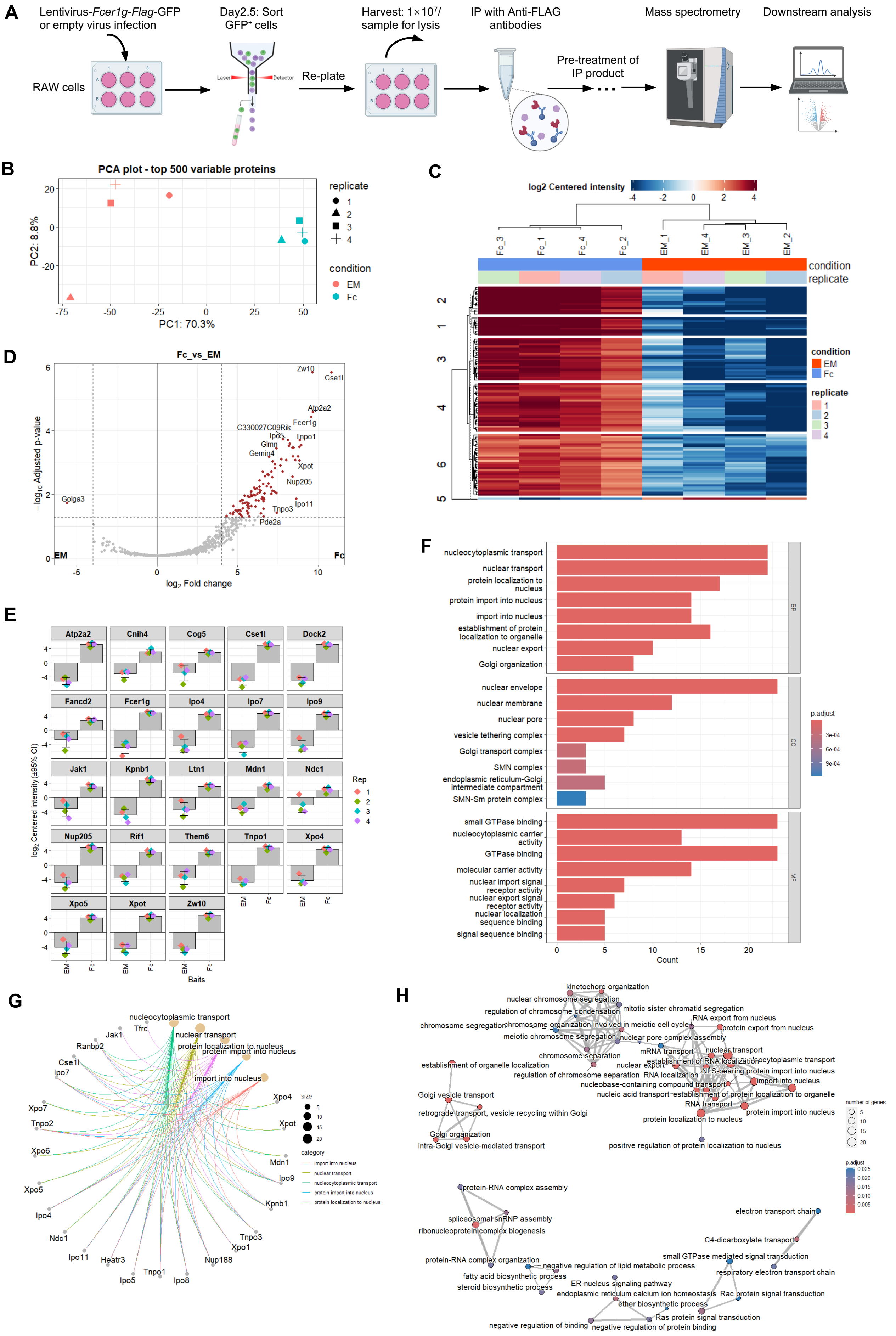

A
Lentivirus-Fcer1g-Flag-GFP or empty virus infection
RAW cells
Day2.5: Sort GFP⁺ cells
Laser
Detector
Re-plate
Harvest: 1×10⁷/ sample for lysis
IP with Anti-FLAG antibodies
Pre-treatment of IP product
Mass spectrometry
Downstream analysis
B
PCA plot - top 500 variable proteins
PC2: 8.8%
PC1: 70.3%
replicate
condition
EM
Fc
C
log2 Centered intensity
Fc_3
Fc_1
Fc_4
Fc_2
EM_1
EM_4
EM_3
EM_2
condition
replicate
D
Fc_vs_EM
−log10 Adjusted p-value
log2 Fold change
Zw10
Cse1l
Atp2a2
Fcer1g
C330027C09Rik
Ipo5
Tnpo1
Glmn
Gemin4
Xpot
Nup205
Ipo11
Tnpo3
Pde2a
Golga3
EM
Fc
E
Atp2a2
Cnih4
Cog5
Cse1l
Dock2
Fancd2
Fcer1g
Ipo4
Ipo7
Ipo9
Jak1
Kpnb1
Ltn1
Mdn1
Ndc1
Nup205
Rif1
Them6
Tnpo1
Xpo4
Xpo5
Xpot
Zw10
log2 Centered intensity(±95% CI)
Rep
Baits
F
nucleocytoplasmic transport
nuclear transport
protein localization to nucleus
protein import into nucleus
import into nucleus
establishment of protein localization to organelle
nuclear export
Golgi organization
nuclear envelope
nuclear membrane
nuclear pore
vesicle tethering complex
Golgi transport complex
SMN complex
endoplasmic reticulum-Golgi intermediate compartment
SMN-Sm protein complex
small GTPase binding
nucleocytoplasmic carrier activity
GTPase binding
molecular carrier activity
nuclear import signal receptor activity
nuclear export signal receptor activity
nuclear localization sequence binding
signal sequence binding
BP
CC
MF
p.adjust
Count
G
nucleocytoplasmic transport
nuclear transport
protein localization to nucleus
protein import into nucleus
import into nucleus
Tfrc
Jak1
Ranbp2
Cse1l
Ipo7
Xpo7
Tnpo2
Xpo6
Xpo5
Ipo4
Ndc1
Ipo11
Heatr3
Tnpo1
Ipo5
Ipo8
Nup188
Xpo1
Tnpo3
Kpnb1
Ipo9
Mdn1
Xpot
Xpo4
size
category
H
kinetochore organization
nuclear chromosome segregation
regulation of chromosome condensation
mitotic sister chromatid segregation
chromosome segregation
chromosome organization involved in meiotic cell cycle
RNA export from nucleus
protein export from nucleus
meiotic chromosome segregation
nuclear pore complex assembly
chromosome separation
mRNA transport
nuclear transport
establishment of organelle localization
nuclear export
establishment of RNA localization
nucleocytoplasmic transport
regulation of chromosome separation
RNA localization
NLS-bearing protein import into nucleus
nucleobase-containing compound transport
import into nucleus
nucleic acid transport
establishment of protein localization to organelle
Golgi vesicle transport
RNA transport
retrograde transport, vesicle recycling within Golgi
protein import into nucleus
protein localization to nucleus
Golgi organization
intra-Golgi vesicle-mediated transport
positive regulation of protein localization to nucleus
number of genes
p.adjust
protein-RNA complex assembly
spliceosomal snRNP assembly
ribonucleoprotein complex biogenesis
electron transport chain
C4-dicarboxylate transport
protein-RNA complex organization
small GTPase mediated signal transduction
negative regulation of lipid metabolic process
respiratory electron transport chain
fatty acid biosynthetic process
steroid biosynthetic process
ER-nucleus signaling pathway
endoplasmic reticulum calcium ion homeostasis
Rac protein signal transduction
ether biosynthetic process
Ras protein signal transduction
negative regulation of binding
negative regulation of protein binding

Supplementary Figure 7. FcεR1γ mediated effector signaling transduction. Related to Fig. 5

A Experimental scheme of IP-MS assay for interaction partners of FcεR1γ (**Methods**, created with BioRender.com).

B The PCA plot showing the enriched top 500 variable proteins by anti-FLAG antibody in IP-MS from the two groups. EM represented RAW cells infected with empty lentivirus, and Fc represented RAW cells infected with lentivirus that express *Fcer1g-flag*. *n*=4 replicates per condition.

C The heatmap of differentially enriched proteins (DEPs) of two groups from (**B**), p.adjust< 0.05 and fold change >4.

D The volcano map showing the DEPs of IP-MS by anti-FLAG antibody from the two groups, the dotted lines represent p.adjust=0.05 or fold change=4.

E The bar plots for the indicated proteins with the data centered.

F The bar plot showing the positively enriched pathways in the Fc group versus EM group in GO. **G** The enrichplot of the proteins that enriched by anti-FLAG from Fc group, p.adjust< 0.05 and fold change >4.

H The enrichment map showing the positively enriched pathways in the Fc group versus EM group in GO.

Figure S8

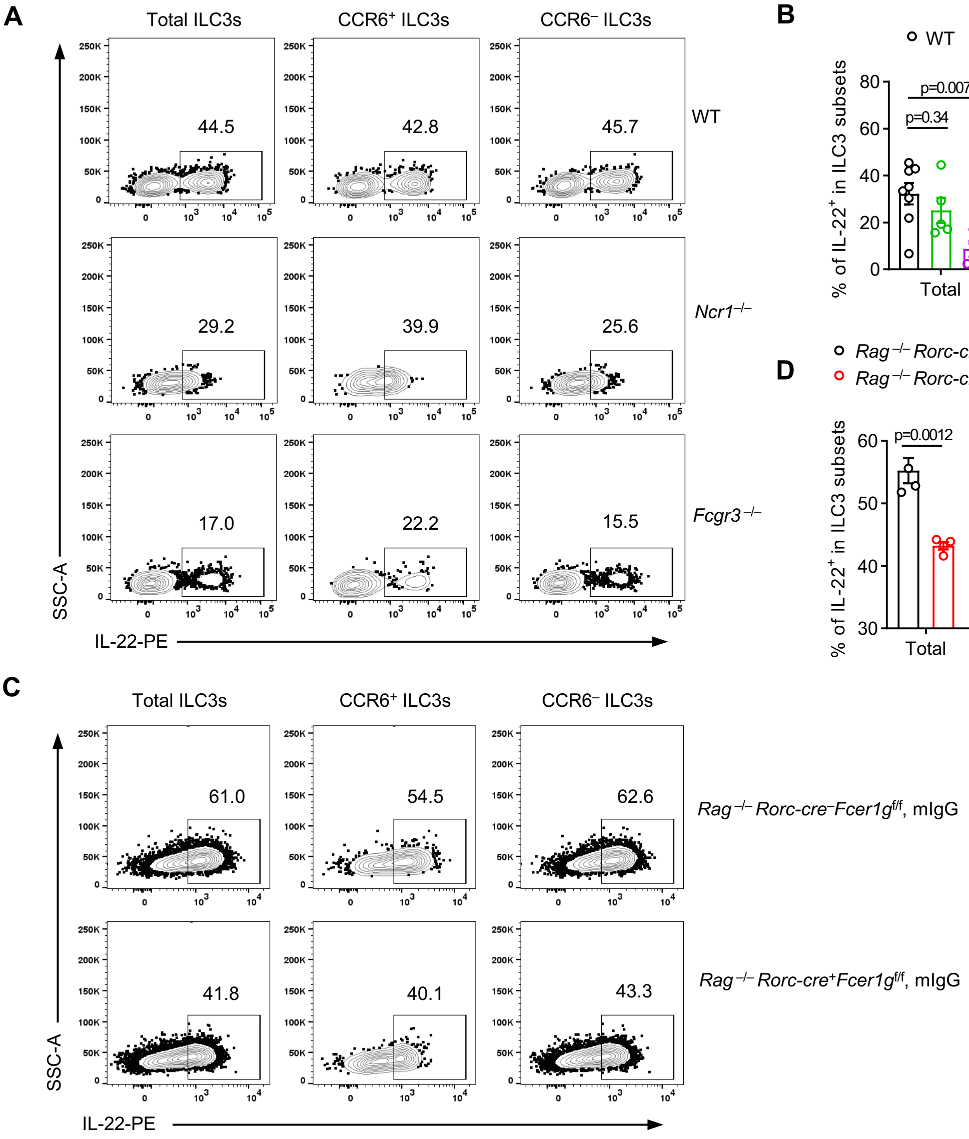

Supplementary Figure 8. FcεR1γ-mediated effector signaling transduction depended on CD16. Related to Fig.7

A-B Representative flow plots showing the intracellular abundance of IL-22 (**A**) in total, CCR6^{+} and CCR6$^{–}$ ILC3s from siLPs of indicated mice on day 5 post the *C. albicans* infection. Quantification of the frequencies of IL-22 (**B**) -expressing cells in each compartment. The cells were stimulated with Cell Stimulation Cocktail, Protein Transport Inhibitor Cocktail and rmIL-23 in complete RPMI medium for 4 hours before staining (**Methods**) (WT, *n*=8 mice; *Ncr1*$^{–/–}$, *n*=5 mice; *Fcgr3*$^{–/–}$, *n*=4 mice).

C-D *Rag1*$^{–/–}$*Rorc-cre*$^{–}$*Fcer1g*$^{f/f}$ and *Rag1*$^{–/–}$*Rorc-cre*$^{+}$*Fcer1g*$^{f/f}$ mice that administration with total mIgG were infected with *C. albicans*. All mice were intravenously infected with 1.5×10^{6} CFU *C. albicans* on day 0 and intraperitoneally injected with 200μg mIgG on day -1 and day 3 (**Methods**). Representative flow plots (**C**) showing the intracellular abundance of IL-22 in total, CCR6^{+} and CCR6$^{–}$ ILC3s from siLPs on day 5 post the infection. The cells were stimulated with Cell Stimulation Cocktail, Protein Transport Inhibitor Cocktail and rmIL-23 in complete RPMI medium for 4 hours before staining (**Methods**). Quantification of the frequencies of IL-22 (**D**) -expressing cells in each compartment. *n*=4 mice per group.

Data are pooled from three (**A, B**) or representative of two (**C, D**) independent experiments shown as the mean ± SEM. Statistical significance was tested by two-tailed *t* test (**B, D**).

Figure S9

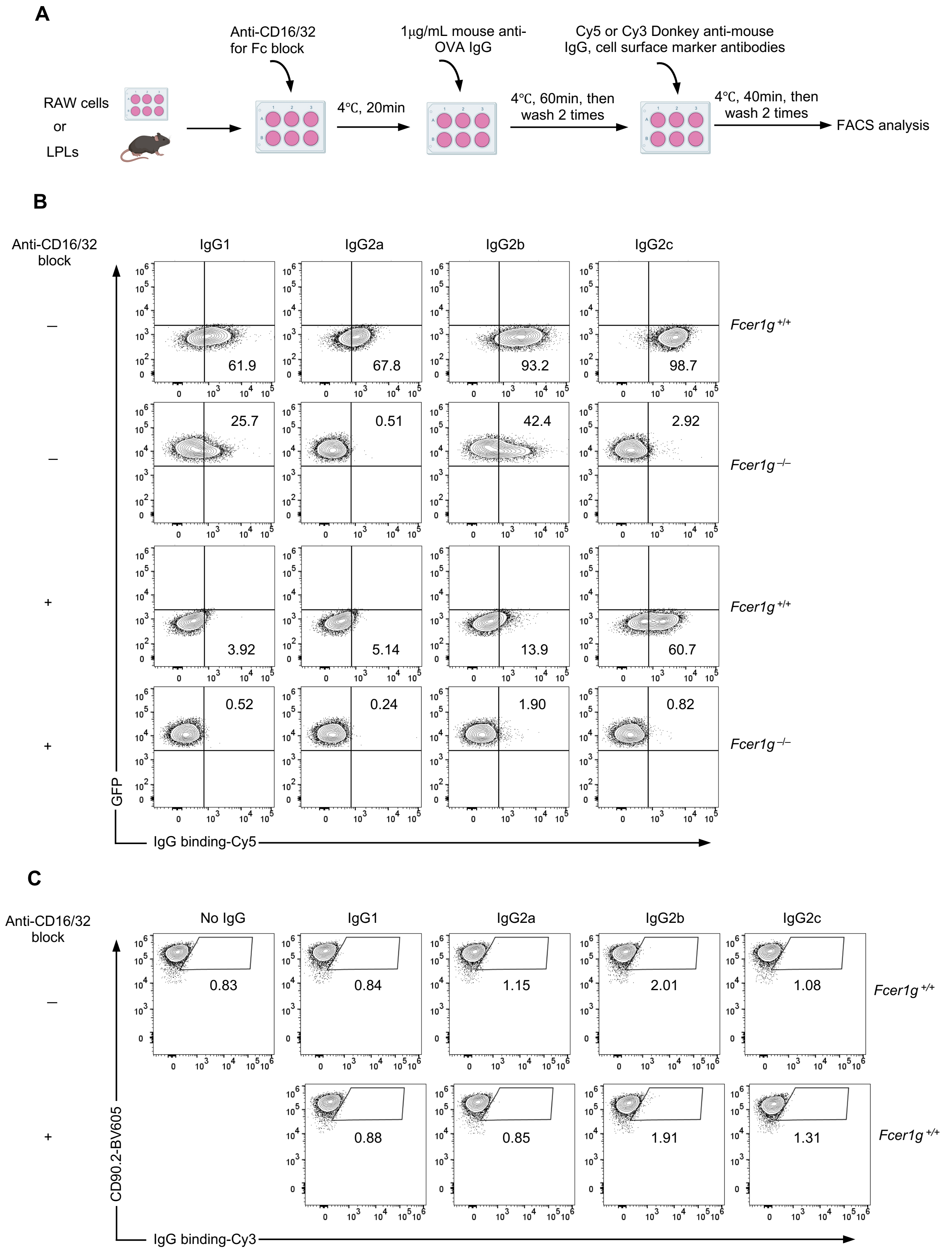

Supplementary Figure 9. The *in vitro* binding of IgG on RAW cells and primary ILC3s.
A Experimental scheme (**Methods**, created with BioRender.com).
B Representative flow plots showing the binding of different IgG isotypes on wild type RAW or *Fcer1g*$^{-/-}$ RAW cells with or without the pre-blocking with anti-Fc receptor antibody.
C Representative flow plots showing the IgG binding on WT ILC3s (Gated on live CD45^{+}Lin^{-}CD90.2^{hi} KLRG1^{-} CD45^{low} cells) with or without Fc block.
Data are representative of two (**B, C**) independent experiments.

Supplementary Table 1 Antibodies used in this study

Antibodies	Source	Catalog number	Dilution	Application
BV421 anti-mouse CCR6(CD196), Clone: 140706	BD Biosciences	564736	1:100	Flow cytometry
BV421 anti-mouse RORgt, Clone: Q31-378	BD Biosciences	562894	1:100	Flow cytometry
BV421 anti-mouse KLRG1, Clone: 2F1/KLRG1	BioLegend	138413/138414	1:200	Flow cytometry
BV510 anti-mouse CD335(NKp46), Clone: 29A1.4	BioLegend	137623	1:200	Flow cytometry
BV510 anti-mouse CD80, Clone: 16-10A1	BioLegend	104741	1:200	Flow cytometry
BV605 anti-mouse CD90.2, Clone: 30-H12	BioLegend	105343	1:200	Flow cytometry
BV605 anti-mouse CD64(FcγRI), Clone: X54-5/7.1	BioLegend	139323	1:200	Flow cytometry
BV785 anti-mouse CD86, Clone: GL-1	BioLegend	105043	1:200	Flow cytometry
eFluor 450 anti-mouse CD16/32, Clone: 93	eBioscience	48-0161-80/48-0161-82	1:200	Flow cytometry
eFluor 450 anti-mouse KLRG1, Clone: 2F1	eBioscience	48-5893-82	1:200	Flow cytometry
eFluor 450 anti-mouse Rat IgG2a, κ, Clone: eBR2a	eBioscience	48-4321-80	1:200	Flow cytometry
FITC anti-mouse FcεRI,γ subunit , polyclonal	Milli-Mark	FCABS400F	1:100	Flow cytometry
FITC anti-mouse KLRG1, Clone: 2F1/KLRG1	BioLegend	138410	1:200	Flow cytometry
FITC anti-mouse Rabbit IgG	eBioscience	11-4614-80	1:200	Flow cytometry
FITC anti-mouse SiglecF(CD170) , Clone: S17007L	BioLegend	155503/155504	1:200	Flow cytometry
FITC anti-mouse TLR2, Clone: 6C2	eBioscience	11-9021-82	1:200	Flow cytometry
Alexa Fluor 488 anti-mouse GATA3, Clone: TWAJ	eBioscience	53-9966-42	1:100	Flow cytometry
Alexa Fluor 488 anti-mouse FcεRIα, Clone: 43525	BioLegend	134330	1:200	Flow cytometry
Alexa Fluor 488 anti-mouse NK1.1, Clone: PK136	BioLegend	108718	1:200	Flow cytometry
Alexa Fluor 488 anti-mouse CD45, Clone: 30-F11	BioLegend	103122	1:200	Flow cytometry
Alexa Fluor 488 anti-mouse IL-17A, Clone: TC11-18H10.1	BioLegend	506910	1:100	Flow cytometry
PerCP/Cy5.5 anti-mouse MHC II (I-A/I-E), Clone: M5/114.15.2	BioLegend	107625/107626	1:100	Flow cytometry
PerCP/Cy5.5 anti-mouse CD335(NKp46), Clone: 29A1.4	BioLegend	137609	1:100	Flow cytometry
Phospho-JAK1 (Tyr1022, Tyr1023) Rabbit Antibody, Clone: 59H4L5	Thermo	700028	1:200	Flow cytometry
Rabbit monoclonal antibody to Phospho-JAK2 (Y1007, Y1008), Clone: E132	abcam	ab32101	1:200	Flow cytometry
Phospho-JAK3 (Tyr981) Polyclonal Antibody	Thermo	PA5-105892	1:200	Flow cytometry
Cy2 AffiniPure Donkey Anti-Rabbit IgG (H+L)	Jackson Immunoresearch	711-225-152	1:500	Flow cytometry
Cy3 AffiniPure Donkey Anti-Mouse IgG (H+L)	Jackson Immunoresearch	715-165-150	1:500	Flow cytometry
Cy5 AffiniPure Donkey Anti-Mouse IgG (H+L)	Jackson Immunoresearch	715-175-151	1:500	Flow cytometry
Mouse Anti-Ovalbumin IgG2a Monoclonal Antibody, Clone: M12E4D5	Chondrex	7095	1:1000	In vitro binding
Mouse Anti-Ovalbumin IgG2b Monoclonal Antibody, Clone: 4B4E6	Chondrex	7096	1:1000	In vitro binding
Mouse Anti-Ovalbumin IgG2c Monoclonal Antibody, Clone: 3E3A9	Chondrex	7109	1:1000	In vitro binding
Mouse Anti-Ovalbumin IgG1 Monoclonal Antibody, Clone: 6C8	abcam	ab17293	1:1000	In vitro binding
PerCP-eFluor 710 anti-mouse CD90.2 (Thy-1.2), Clone: 30-H12	eBioscience	46-0903-82	1:200	Flow cytometry

Supplementary Table 1 Antibodies used in this study (Continued)

Antibodies	Source	Catalog number	Dilution	Application
PerCP-eFluor 710 anti-mouse CD4, Clone: GK1.5	Invitrogen	46-0041-82	1:200	Flow cytometry
PE anti-mouse KLRG1, Clone: 2F1/KLRG1	BioLegend	138408	1:200	Flow cytometry
PE anti-mouse CD16, clone: S17014E	BioLegend	158003	1:200	Flow cytometry
PE anti-mouse Dectin-1, Clone: RH1	BioLegend	144303	1:200	Flow cytometry
PE anti-mouse Rat IgG2a, κ, Clone: R35-95	BD Biosciences	553930	1:100	Flow cytometry
PE anti-mouse RORgt, Clone: B2D	eBioscience	12-6981-82	1:100	Flow cytometry
PE anti-mouse IL-22, Clone: 1H8PWSR	eBioscience	12-7221-82	1:100	Flow cytometry
PE anti-mouse Foxp3, Clone: FJK-16s	eBioscience	12-5773-82	1:100	Flow cytometry
PE anti-mouse CD170 (Siglec F), Clone: 1RNM44N	Invitrogen	12-1702-82	1:200	Flow cytometry
PE/Cyanine7 anti-mouse CD127(IL-7R), Clone: A7R34	BioLegend	135014	1:200	Flow cytometry
PE/Cyanine7 anti-mouse CD3, Clone: 17A2	BioLegend	100220	1:200	Flow cytometry
PE/Cyanine7 anti-mouse CD19, Clone: eBio1D3	eBioscience	25-0193-82	1:200	Flow cytometry
PE/Cyanine7 anti-mouse CD335(NKp46), Clone: 29A1.4	eBioscience	25-3351-80/25-3351-82	1:200	Flow cytometry
PE/Cyanine7 anti-mouse T-bet, Clone: eBio4B10 (4B10)	BioLegend	25-5825-82	1:100	Flow cytometry
APC anti-mouse CD3e, Clone: 145-2C11	BioLegend	100312	1:200	Flow cytometry
APC anti-mouse CD19, Clone: 6D5	BioLegend	115512	1:200	Flow cytometry
APC anti-mouse Ly-6G/Ly-6C (Gr1), Clone: RB6-8C5	BioLegend	108412	1:200	Flow cytometry
APC anti-mouse FcεRIα, Clone: MAR-1	BioLegend	134316	1:200	Flow cytometry
APC anti-mouse TCRab, Clone: H57-597	BioLegend	109212	1:200	Flow cytometry
APC anti-mouse TCR γ/δ, Clone: GL3	BioLegend	118116	1:200	Flow cytometry
APC anti-mouse CD11b, Clone: M1/70	BioLegend	101212	1:200	Flow cytometry
APC anti-mouse CD11c, Clone: N418	BioLegend	117310	1:200	Flow cytometry
APC anti-mouse CD5, Clone: 53-7.3	BioLegend	100626	1:200	Flow cytometry
Alexa Fluor 647 anti-mouse KLRG1, Clone: 2F1	eBioscience	51-5893-82	1:200	Flow cytometry
Alexa Fluor 647 anti-mouse SiglecF(CD170) , Clone: E50-2440	BD Biosciences	562680	1:200	Flow cytometry
Alexa Fluor 700 anti-mouse CD45, Clone: 30-F11	BD Biosciences	560510	1:200	Flow cytometry
Purified anti-mouse CD16/CD32, Clone: 2.4G2	BD Biosciences	553142	1:200	Flow cytometry
Purified anti-mouse FcεRI,γ subunit polyclonal	Upstate	06-727	1:100	Flow cytometry
Purified mouse anti-FLAG, clone: M2	Sigma-Aldrich	F1804	1:250	Immunoprecipitation
TotalSeq™-B0301 anti-mouse Hashtag 1 Antibody	BioLegend	155831	1:400	scRNAseq
TotalSeq™-B0302 anti-mouse Hashtag 2 Antibody	BioLegend	155833	1:400	scRNAseq
TotalSeq™-B0303 anti-mouse Hashtag 3 Antibody	BioLegend	155835	1:400	scRNAseq
TotalSeq™-B0304 anti-mouse Hashtag 4 Antibody	BioLegend	155837	1:400	scRNAseq
TotalSeq™-B0305 anti-mouse Hashtag 5 Antibody	BioLegend	155839	1:400	scRNAseq
TotalSeq™-B0306 anti-mouse Hashtag 6 Antibody	BioLegend	155841	1:400	scRNAseq

Supplementary Table 2 Oligonucleotides used in this study

**Oligonucleotide**	**Source**	**Sequence(5’-3’)**	**Application**
m*Il17a*-F	Sangon Biotech	tctgtgtctctgatgctgttg	Primers for qPCR
m*Il17a*-R	Sangon Biotech	tatcagggtcttcattgcgg	Primers for qPCR
m*Il22*-F	Sangon Biotech	tgacacttgtgcgatctctg	Primers for qPCR
m*Il22*-R	Sangon Biotech	actgatccttagcactgactcc	Primers for qPCR
m*Tnfa*-F	Sangon Biotech	ccctccagaaaagacaccatg	Primers for qPCR
m*Tnfa*-R	Sangon Biotech	caggcttgtcactcgaattttg	Primers for qPCR
m*Fcgr1*-F	Sangon Biotech	aagacaccgctacacatctg	Primers for qPCR
m*Fcgr1*-R	Sangon Biotech	tctttccgcccttgctatatg	Primers for qPCR
m*Fcgr4*-F	Sangon Biotech	ccaacagctctggtacttacag	Primers for qPCR
m*Fcgr4*-R	Sangon Biotech	tccttaactctggcactttgg	Primers for qPCR
m*Fcgr2b*-F	Sangon Biotech	tgtcaccatcactgtccaag	Primers for qPCR
mFcgr2b-R	Sangon Biotech	tgtactcacctacttcctctgg	Primers for qPCR
m*Ncr1*-F	Sangon Biotech	gggatctacacctgcttctatc	Primers for qPCR
m*Ncr1*-R	Sangon Biotech	tgggaaactctgcttggatg	Primers for qPCR
m*Fcgr3*-F	Sangon Biotech	tcttgaatgactttggacaccc	Primers for qPCR
m*Fcgr3*-R	Sangon Biotech	ttgacaccgatattctccactg	Primers for qPCR
m*Gapdh*-F	Sangon Biotech	aatggtgaaggtcggtgtg	Primers for qPCR
m*Gapdh*-R	Sangon Biotech	acaagcttcccattctcgg	Primers for qPCR
m*Fcer1g*(Exon2,3,5)-F	Sangon Biotech	ctcaagatccaggtccgaaag	Primers for qPCR
m*Fcer1g*(Exon2,3,5)-R	Sangon Biotech	gggaaaagaatgcagccaag	Primers for qPCR
m*Actb*-F	Sangon Biotech	agctgtgctatgttgctctag	Primers for qPCR
m*Actb*-R	Sangon Biotech	aggtctttacggatgtcaacg	Primers for qPCR
mFcer1g-L19A-F	Tsingke	gccgccgcaggagagccgcagctctgctatatcctgg atgc	Primers for mutated *Fcer1g*
mFcer1g-L19A-R	Tsingke	gctgcggctctcctgcggcggctgcttgttccaccaaaa ggagc	Primers for mutated *Fcer1g*
mFcer1g-G20A-F	Tsingke	gccgccctggcagagccgcagctctgctatatcctgga tgctg	Primers for mutated *Fcer1g*
mFcer1g-G20A-R	Tsingke	gctgcggctctgccagggcggctgcttgttccaccaaa aggag	Primers for mutated *Fcer1g*
mFcer1g-E21A-F	Tsingke	gccgccctgggagcaccgcagctctgctatatcc tggatgctg	Primers for mutated *Fcer1g*
mFcer1g-E21A-R	Tsingke	gctgcggtgctcccagggcggctgcttgttccac caaaaggag	Primers for mutated *Fcer1g*
mFcer1g-P22A-F	Tsingke	gccgccctgggagaggcacagctctgctatatcc tggatgctg	Primers for mutated *Fcer1g*
mFcer1g-P22A-R	Tsingke	gctgtgcctctcccagggcggctgcttgttccacc aaaaggag	Primers for mutated *Fcer1g*
mFcer1g-Q23A-F	Tsingke	gccgccctgggagagccggcactctgctatatcc tggatgctg	Primers for mutated *Fcer1g*
mFcer1g-Q23A-R	Tsingke	cagagtgccggctctcccagggcggctgcttgtt ccaccaaaaggag	Primers for mutated *Fcer1g*
mFcer1g-L24A-F	Tsingke	ccgcaggcatgctatatcctggatgctgtcct	Primers for mutated *Fcer1g*
mFcer1g-L24A-R	Tsingke	atatagcatgcctgcggctctcccagggc	Primers for mutated *Fcer1g*
mFcer1g-C25A-F	Tsingke	gcagctcgcatatatcctggatgctgtcctgtttt	Primers for mutated *Fcer1g*
mFcer1g-C25A-R	Tsingke	ggatatatgcgagctgcggctctcccag	Primers for mutated *Fcer1g*
mFcer1g-Y26A-F	Tsingke	tctgcgcaatcctggatgctgtcctgtttt	Primers for mutated *Fcer1g*
mFcer1g-Y26A-R	Tsingke	atccaggattgcgcagagctgcggctctcc	Primers for mutated *Fcer1g*

Supplementary Table 2 Oligonucleotides used in this study (Continued)

**Oligonucleotide**	**Source**	**Sequence(5'-3')**	**Application**
mFcer1g-I27A-F	Tsingke	ctgctatgcactggatgctgtcctgtttttgtat	Primers for mutated *Fcer1g*
mFcer1g-I27A-R	Tsingke	catccagtgcatagcagagctgcggctctcc	Primers for mutated *Fcer1g*
mFcer1g-L28A-F	Tsingke	ctatatcgcagatgctgtcctgtttttgtatggt	Primers for mutated *Fcer1g*
mFcer1g-L28A-R	Tsingke	cagcatctgcgatatagcagagctgcggctct	Primers for mutated *Fcer1g*
mFcer1g-D29A-F	Tsingke	atatcctggcagctgtcctgtttttgtatggtattg	Primers for mutated *Fcer1g*
mFcer1g-D29A-R	Tsingke	gacagctgccaggatatagcagagctgcgg	Primers for mutated *Fcer1g*
mFcer1g-V31A-F	Tsingke	ggatgctgcactgtttttgtatggtattgtccttacc	Primers for mutated *Fcer1g*
mFcer1g-V31A-R	Tsingke	aaaacagtgcagcatccaggatatagcagagctg	Primers for mutated *Fcer1g*
mFcer1g-L32A-F	Tsingke	ggatgctgtcgcatttttgtatggtattgtccttaccct	Primers for mutated *Fcer1g*
mFcer1g-L32A-R	Tsingke	aaaatgcgacagcatccaggatatagcagag	Primers for mutated *Fcer1g*
mFcer1g-F33A-F	Tsingke	tgtcctggcattgtatggtattgtccttaccctactc	Primers for mutated *Fcer1g*
mFcer1g-F33A-R	Tsingke	catacaatgccaggacagcatccaggatatagc	Primers for mutated *Fcer1g*
mFcer1g-L34A-F	Tsingke	gtcctgtttgcatatggtattgtccttaccctactctactg	Primers for mutated *Fcer1g*
mFcer1g-L34A-R	Tsingke	ccatatgcaaacaggacagcatccaggatatag	Primers for mutated *Fcer1g*
mFcer1g-Y35A-F	Tsingke	cctgtttttggcaggtattgtccttaccctactctactgtc	Primers for mutated *Fcer1g*
mFcer1g-Y35A-R	Tsingke	tacctgccaaaaacaggacagcatccagg	Primers for mutated *Fcer1g*
mFcer1g-G36A-F	Tsingke	tgcaattgtccttaccctactctactgtcga	Primers for mutated *Fcer1g*
mFcer1g-G36A-R	Tsingke	gggtaaggacaattgcatacaaaaacaggacagcatcc ag	Primers for mutated *Fcer1g*
mFcer1g-I37A-F	Tsingke	gtatggtgcagtccttaccctactctactgtcgactc	Primers for mutated *Fcer1g*
mFcer1g-I37A-R	Tsingke	taaggactgcaccatacaaaaacaggacagcatc	Primers for mutated *Fcer1g*
mFcer1g-V38A-F	Tsingke	tggtattgcacttaccctactctactgtcgactcaag	Primers for mutated *Fcer1g*
mFcer1g-V38A-R	Tsingke	gggtaagtgcaataccatacaaaaacaggacagca	Primers for mutated *Fcer1g*
mFcer1g-L39A-F	Tsingke	tattgtcgcaaccctactctactgtcgactcaagatc	Primers for mutated *Fcer1g*
mFcer1g-L39A-R	Tsingke	gtagggttgcgacaataccatacaaaaacaggacagc	Primers for mutated *Fcer1g*
mFcer1g-T40A-F	Tsingke	tgtccttgcactactctactgtcgactcaagatccag	Primers for mutated *Fcer1g*
mFcer1g-T40A-R	Tsingke	agagtagtgcaaggacaataccatacaaaaacagga	Primers for mutated *Fcer1g*
mFcer1g-L41A-F	Tsingke	ccttaccgcactctactgtcgactcaagatccagg	Primers for mutated *Fcer1g*
mFcer1g-L41A-R	Tsingke	agtagagtgcggtaaggacaataccatacaaaaacag	Primers for mutated *Fcer1g*
mFcer1g-L42A-F	Tsingke	taccctagcatactgtcgactcaagatccaggtc	Primers for mutated *Fcer1g*
mFcer1g-L42A-R	Tsingke	gacagtatgctagggtaaggacaataccatacaaaaa	Primers for mutated *Fcer1g*
mFcer1g-Y43A-F	Tsingke	ctactcgcatgtcgactcaagatccaggtcc	Primers for mutated *Fcer1g*
mFcer1g-Y43A-R	Tsingke	gtcgacatgcgagtagggtaaggacaataccatacaa	Primers for mutated *Fcer1g*
mFcer1g-C44A-F	Tsingke	actctacgcacgactcaagatccaggtccg	Primers for mutated *Fcer1g*
mFcer1g-C44A-R	Tsingke	tgagtcgtgcgtagagtagggtaaggacaataccatac a	Primers for mutated *Fcer1g*
sgRNA-mFcer1g-Oligo-1	Sangon Biotech	caccggtcgacagtagagtagggta	sgRNA for m*Fcer1g* knockout
sgRNA-mFcer1g-Oligo-2	Sangon Biotech	aaactaccctactctactgtcgacc	sgRNA for m*Fcer1g* knockout
