# Supplementary material for: Antibody Fc-receptor FcεR1γ stabilizes cell surface receptors in group 3 innate lymphoid cells and promotes anti-infection immunity
Source: Nat Commun. 2024 Jul 16;15:5981. doi: 10.1038/s41467-024-50266-4 (PMC11252441; doi:10.1038/s41467-024-50266-4)
Supplement: Supplementary file 3 — Reporting Summary [file 41467_2024_50266_MOESM3_ESM.pdf]

nature portfolio

Corresponding author(s): Heping Xu, Chao Huang

Last updated by author(s): 2024-4-28

Reporting Summary

Nature Portfolio wishes to improve the reproducibility of the work that we publish. This form provides structure for consistency and transparency in reporting. For further information on Nature Portfolio policies, see our Editorial Policies and the Editorial Policy Checklist.

Statistics

For all statistical analyses, confirm that the following items are present in the figure legend, table legend, main text, or Methods section.

n/a	Confirmed	
☐	☒	The exact sample size (*n*) for each experimental group/condition, given as a discrete number and unit of measurement
☐	☒	A statement on whether measurements were taken from distinct samples or whether the same sample was measured repeatedly
☐	☒	The statistical test(s) used AND whether they are one- or two-sided<br>*Only common tests should be described solely by name; describe more complex techniques in the Methods section.*
☐	☒	A description of all covariates tested
☐	☒	A description of any assumptions or corrections, such as tests of normality and adjustment for multiple comparisons
☐	☒	A full description of the statistical parameters including central tendency (e.g. means) or other basic estimates (e.g. regression coefficient) AND variation (e.g. standard deviation) or associated estimates of uncertainty (e.g. confidence intervals)
☐	☒	For null hypothesis testing, the test statistic (e.g. *F*, *t*, *r*) with confidence intervals, effect sizes, degrees of freedom and *P* value noted<br>*Give P values as exact values whenever suitable.*
☒	☐	For Bayesian analysis, information on the choice of priors and Markov chain Monte Carlo settings
☒	☐	For hierarchical and complex designs, identification of the appropriate level for tests and full reporting of outcomes
☒	☐	Estimates of effect sizes (e.g. Cohen's *d*, Pearson's *r*), indicating how they were calculated

Our web collection on statistics for biologists contains articles on many of the points above.

Software and code

Policy information about availability of computer code

Data collection	FACS data were collected by CytExpert software with CytoFLEX (Beckman Coulter), or BD FACSDiva™ Software with LSR Fortessa (BD), FACSAria Fusion (BD) or FACSAira III (BD). Quantitative PCR data were collected on a Qtower384G System (Jena). Bioluminescence imaging (BLI) data were acquired by PhotoAcquisition (Biospace Lab) software. Mass Spectrometry data were analyzed by Orbitrap Exploris™ 480 (Thermo). The Bulk RNA-seq libraries were sequenced (150bp×2) with HiSeq X (Illumina). The scRNA-seq libraries were sequenced (150bp×2) with NovaSeq (Illumina).

Data analysis	FACS data were analyzed by FlowJo software v10.0.7 (TreeStar). Bioluminescence imaging (BLI) data were analyzed by M3Vision (Biospace Lab) software. The raw Mass Spectrometry data were processed by MaxQuant and downstream analysis such as data preparation, filtering, variance normalization and imputation of missing values, as well as statistical testing was processed with the DEP package (v1.25.0) in R.<br>Raw fastq files for bulk-RNA-seq were trimmed using cutadapt (v2.10) and trimmed reads were aligned to the mouse genome/transcriptome (GENCODE GRCm38 vM25, mm10) using STAR (v2.7.5c) with 'twopassModeBasic', expression abundances were estimated (expected-counts and TPM) using RSEM (v1.3.3). The counts matrix output from gene-results were processed with the edgeR package (v3.32.0) in R to analyze differential gene expression with default parameters. GSEA(v4.2.1) were performed using the official tool with filtered edgeR (v3.32.0) normalized (TMM) expression matrix, and results were replotted in R.<br>For scRNA-seq data processing， reads demultiplexing, alignment and unique molecular identifiers (UMIs) counting/collapsing were using the CellRanger toolkit (v6.1.1, 10x Genomics). Main downstream analysis like 'Demultiplexing with hashtag oligos (HTOs)', clustering and filtering were performed in R (v4.0.3)/Rstudio with Seurat (v4.1.0).<br>GraphPad Prism 7.00 software was used for all statistical analysis (except for RNA sequencing data or MS data). Survival curves were analyzed according to the Kaplan-Meier estimator, and the difference between two groups was determined by the log-rank (Mantel-Cox) test. Statistical differences for other experiments were determined by Mann-Whitney U-test or unpaired Student's t test as indicated.

For manuscripts utilizing custom algorithms or software that are central to the research but not yet described in published literature, software must be made available to editors and reviewers. We strongly encourage code deposition in a community repository (e.g. GitHub). See the Nature Portfolio guidelines for submitting code & software for further information.

Data

Policy information about availability of data

All manuscripts must include a data availability statement. This statement should provide the following information, where applicable:

- Accession codes, unique identifiers, or web links for publicly available datasets
- A description of any restrictions on data availability
- For clinical datasets or third party data, please ensure that the statement adheres to our policy

The transcriptomic sequencing data are available at Gene Expression Omnibus (GEO) under accession number (GSE256409). The mass spectrometry proteomics data have been deposited to the ProteomeXchange Consortium via the iProX partner repository under the dataset identifier PXD049222. All other data are available in the manuscript or the supplementary materials.

Research involving human participants, their data, or biological material

Policy information about studies with human participants or human data. See also policy information about sex, gender (identity/presentation), and sexual orientation and race, ethnicity and racism.

Reporting on sex and gender	No human participants in our study
Reporting on race, ethnicity, or other socially relevant groupings	N/A. No human participants in our study
Population characteristics	N/A. No human participants in our study
Recruitment	N/A. No human participants in our study
Ethics oversight	N/A.No human participants in our study

Note that full information on the approval of the study protocol must also be provided in the manuscript.

Field-specific reporting

Please select the one below that is the best fit for your research. If you are not sure, read the appropriate sections before making your selection.

☒ Life sciences ☐ Behavioural & social sciences ☐ Ecological, evolutionary & environmental sciences

For a reference copy of the document with all sections, see nature.com/documents/nr-reporting-summary-flat.pdf

Life sciences study design

All studies must disclose on these points even when the disclosure is negative.

Sample size	Sample sizes were chosen based on each experiment being technically feasible from a work flow standpoint while also providing a reasonable number of replicates to be confident in the results.
Data exclusions	All data were included except when the following technical reasons were applicable: if the preparation had a technical error, if no sample was recovered.
Replication	All findings reported were reliably reproduced. The replicate number is always clearly stated in the legends.

Randomization

Sex-matched and age-matched animals were randomly assigned to experimental arms. Littermates were used where possible. Samples were allocated to groups according to genotype.

Blinding

Animals were grouped unblinded, but investigators were blinded for most of the qualification experiments. Histological analysis were performed by a laboratory blinded to the experimental design.

Reporting for specific materials, systems and methods

We require information from authors about some types of materials, experimental systems and methods used in many studies. Here, indicate whether each material, system or method listed is relevant to your study. If you are not sure if a list item applies to your research, read the appropriate section before selecting a response.

Materials & experimental systems

n/a	Involved in the study	
☐	☒	Antibodies
☐	☒	Eukaryotic cell lines
☒	☐	Palaeontology and archaeology
☐	☒	Animals and other organisms
☒	☐	Clinical data
☒	☐	Dual use research of concern
☒	☐	Plants

Methods

n/a	Involved in the study	
☒	☐	ChIP-seq
☐	☒	Flow cytometry
☒	☐	MRI-based neuroimaging

Antibodies

Antibodies used

BV421 anti-mouse CCR6(CD196), Clone: 140706, BD Biosciences, Cat# 564736.
BV421 anti-mouse RORgt, Clone: Q31-378,BD Biosciences, Cat# 562894
BV421 anti-mouse KLRG1, Clone: 2F1/KLRG1, BioLegend,Cat# 138413/138414
BV510 anti-mouse CD335(NKp46), Clone: 29A1.4 , BioLegend,Cat# 137623
BV510 anti-mouse CD80, Clone: 16-10A1 BioLegend,Cat# 104741
BV605 anti-mouse CD90.2, Clone: 30-H12,BioLegend, Cat# 105343
BV605 anti-mouse CD64(FcγRI), Clone: X54-5/7.1, BioLegend, Cat# 139323
BV785 anti-mouse CD86, Clone: GL-1,BioLegend, Cat# 105043
eFluor 450 anti-mouse CD16/32, Clone: 93, eBioscience, Cat# 48-0161-80/48-0161-82
eFluor 450 anti-mouse KLRG1, Clone: 2F1, eBioscience, Cat# 48-5893-82
eFluor 450 anti-mouse Rat IgG2a, κ, Clone: eBR2a, eBioscience, Cat# 48-4321-80
FITC anti-mouse FcεRI,γ subunit , polyclonal, Milli-Mark, Cat# FCABS400F
FITC anti-mouse KLRG1, Clone: 2F1/KLRG1, BioLegend,Cat# 138410
FITC anti-mouse Rabbit IgG, eBioscience, Cat# 11-4614-80
FITC anti-mouse SiglecF(CD170) , Clone: S17007L, BioLegend,Cat# 155503/155504
FITC anti-mouse TLR2, Clone: 6C2, eBioscience, Cat# 11-9021-82
Alexa Fluor 488 anti-mouse GATA3, Clone: TWAJ, eBioscience, Cat# 53-9966-42
Alexa Fluor 488 anti-mouse FcεRIα, Clone: 43525, BioLegend,Cat# 134330
Alexa Fluor 488 anti-mouse NK1.1, Clone: PK136, BioLegend,Cat# 108718
Alexa Fluor 488 anti-mouse CD45, Clone: 30-F11, BioLegend,Cat# 103122
Alexa Fluor 488 anti-mouse IL-17A, Clone: TC11-18H10.1, BioLegend,Cat# 506910
PerCP/Cy5.5 anti-mouse MHC II (I-A/I-E), Clone: M5/114.15.2, BioLegend,Cat# 107625/107626
PerCP/Cy5.5 anti-mouse CD335(NKp46), Clone: 29A1.4, BioLegend,Cat# 137609
PerCP-eFluor 710 anti-mouse CD90.2 (Thy-1.2), Clone: 30-H12, eBioscience, Cat# 46-0903-82
PerCP-eFluor 710 anti-mouse CD4, Clone: GK1.5, Invitrogen, Cat# 46-0041-82
PerCP-eFluor 710 anti-mouse Rat IgG2a κ, Clone: eBR2a, eBioscience, Cat# 46-4321-80
PE anti-mouse KLRG1, Clone: 2F1/KLRG1, BioLegend,Cat# 138408
PE anti-mouse CD16, clone: S17014E, BioLegend,Cat# 158003
PE anti-mouse Dectin-1, Clone: RH1, BioLegend,Cat# 144303
PE anti-mouse Rat IgG2a, κ, Clone: R35-95, BD Biosciences, Cat# 553930
PE anti-mouse RORgt, Clone: B2D, eBioscience, Cat# 12-6981-82
PE anti-mouse IL-22, Clone: 1H8PWSR, eBioscience, Cat# 12-7221-82
PE anti-mouse Foxp3, Clone: FJK-16s ,eBioscience, Cat# 12-5773-82
PE anti-mouse CD170 (Siglec F), Clone: 1RNM44N, Invitrogen, Cat# 12-1702-82
PE/Cyanine7 anti-mouse CD127(IL-7R), Clone: A7R34, BioLegend,Cat# 135014
PE/Cyanine7 anti-mouse CD3, Clone: 17A2, BioLegend,Cat# 100220
PE/Cyanine7 anti-mouse CD19, Clone: eBio1D3, eBioscience, Cat# 25-0193-82
PE/Cyanine7 anti-mouse CD335(NKp46), Clone: 29A1.4, eBioscience, Cat# 25-3351-80/25-3351-82
PE/Cyanine7 anti-mouse T-bet, Clone: eBio4B10 (4B10), BioLegend,Cat# 25-5825-82
APC anti-mouse CD3e, Clone: 145-2C11, BioLegend,Cat# 100312
APC anti-mouse CD19, Clone: 6D5, BioLegend,Cat# 115512
APC anti-mouse Ly-6G/Ly-6C (Gr1), Clone: RB6-8C5, BioLegend,Cat# 108412
APC anti-mouse FcεRIα, Clone: MAR-1, BioLegend,Cat# 134316
APC anti-mouse TCRab, Clone: H57-597, BioLegend,Cat# 109212

APC anti-mouse TCR γ/δ, Clone: GL3 ,BioLegend, Cat# 118116
APC anti-mouse CD11b, Clone: M1/70, BioLegend,Cat# 101212
APC anti-mouse CD11c, Clone: N418 ,BioLegend, Cat# 117310
APC anti-mouse CD5, Clone: 53-7.3,BioLegend, Cat# 100626
Alexa Fluor 647 anti-mouse KLRG1, Clone: 2F1, eBioscience, Cat# 51-5893-82
Alexa Fluor 647 anti-mouse SiglecF(CD170) , Clone: E50-2440, BD Biosciences, Cat# 562680
Alexa Fluor 700 anti-mouse CD45, Clone: 30-F11, BD Biosciences, Cat# 560510
Purified anti-mouse CD16/CD32, Clone: 2.4G2, BD Biosciences, Cat# 553142
Purified anti-mouse FcεRI,γ subunit polyclonal, Upstate, Cat# 06-727
Purified mouse anti-FLAG, clone: M2, Sigma-Aldrich, Cat# F1804
TotalSeq™-B0301 anti-mouse Hashtag 1 Antibody, BioLegend,Cat# 155831
TotalSeq™-B0302 anti-mouse Hashtag 2 Antibody, BioLegend,Cat# 155833
TotalSeq™-B0303 anti-mouse Hashtag 3 Antibody, BioLegend,Cat# 155835
TotalSeq™-B0304 anti-mouse Hashtag 4 Antibody, BioLegend,Cat# 155837
TotalSeq™-B0305 anti-mouse Hashtag 5 Antibody, BioLegend, Cat# 155839
TotalSeq™-B0306 anti-mouse Hashtag 6 Antibody,BioLegend, Cat# 155841
Phospho-JAK1 (Tyr1022, Tyr1023) Rabbit Antibody, Clone: 59H4L5 , Thermo, Cat# 700028
Rabbit monoclonal antibody to Phospho-JAK2 (Y1007, Y1008), Clone: E132, abcam , Cat# ab32101
Phospho-JAK3 (Tyr981) Polyclonal Antibody , Thermo , Cat# PA5-105892
Cy2 AffiniPure Donkey Anti-Rabbit IgG (H+L) , Jackson Immunoresearch, Cat# 711-225-152
Cy3 AffiniPure Donkey Anti-Mouse IgG (H+L), Jackson Immunoresearch , Cat# 715-165-150
Cy5 AffiniPure Donkey Anti-Mouse IgG (H+L) , Jackson Immunoresearch , Cat# 715-175-151
Mouse Anti-Ovalbumin IgG2a Monoclonal Antibody, Clone: M12E4D5 , Chondrex, Cat# 7095
Mouse Anti-Ovalbumin IgG2b Monoclonal Antibody, Clone: 4B4E6, Chondrex , Cat# 7096
Mouse Anti-Ovalbumin IgG2c Monoclonal Antibody, Clone: 3E3A9, Chondrex , Cat# 7109
Mouse Anti-Ovalbumin IgG1 Monoclonal Antibody, Clone: 6C8 , abcam, Cat# ab17293

Validation

All antibodies used are well-established antibodies. Staining patterns were consistent with the manufacturer product information as well as published data.

Eukaryotic cell lines

Policy information about cell lines and Sex and Gender in Research

Cell line source(s)

HEK293T is purchased from ATCC (CRL-3216). RAW 264.7 is purchased from ATCC (TIB-71)

Authentication

The cell lines were authenticated based on morphology comparison with ATCC culture collection database images

Mycoplasma contamination

Cells have been routinely tested for mycoplasma and they were negative

Commonly misidentified lines
(See ICLAC register)

No

Animals and other research organisms

Policy information about studies involving animals; ARRIVE guidelines recommended for reporting animal research, and Sex and Gender in Research

Laboratory animals

C57BL/6J (Jax 000664), Rorc-cre mice (B6.FVB-Tg(Rorc-cre)1Litt/J, Jax 022791) and Rag1 KO mice (B6.129S7-Rag1tm1Mom/J, Jax 002216) were obtained from the Jackson Laboratory. Fcer1g floxtm1c mice were produced by crossing Fcer1gtm1a(KOMP) allele from NIH Knockout Mouse Project (KOMP) with an Flp transgenic mouse strain, removing the FRT-flanked knockout-first cassette. Rorc-cre Fcer1g flox mice were generated by mating Rorc-cre mice with Fcer1g floxtm1c mice. Rag1–/– Rorc-cre Fcer1g flox mice were generated by mating Rag1 KO mice with Rorc-cre Fcer1g flox mice. Fcer1g KO (Fcer1g Germline KO) mice were purchased from Shanghai Model Organisms Center. Ncr1 KO mice and Fcgr3 KO mice were generated at the Center for Excellence in Molecular Cell Science, Chinese Academy of Sciences. All mice were housed in specific-pathogen-free conditions at 22-24C with humidity of 40%–70% and a light/dark cycle of 12 hours, and were used and maintained in accordance with the Institutional Animal Care and Use Committee Guidelines of Westlake University.

Wild animals

No wild animal was used in this study

Reporting on sex

Male and female mice have been used in our study

Field-collected samples

None field-collected sample was used in our study

Ethics oversight

All mice were housed in specific-pathogen-free conditions at 22-24C with humidity of 40%–70% and a light/dark cycle of 12 hours, and were used and maintained in accordance with the Institutional Animal Care and Use Committee Guidelines of Westlake University.

Note that full information on the approval of the study protocol must also be provided in the manuscript.

Plants

Seed stocks

We did not use any plants in our study

Novel plant genotypes

We did not use any plants in our study

Authentication

We did not use any plants in our study

Flow Cytometry

Plots

Confirm that:

☒ The axis labels state the marker and fluorochrome used (e.g. CD4-FITC).

☒ The axis scales are clearly visible. Include numbers along axes only for bottom left plot of group (a 'group' is an analysis of identical markers).

☒ All plots are contour plots with outliers or pseudocolor plots.

☒ A numerical value for number of cells or percentage (with statistics) is provided.

Methodology

Sample preparation

Isolation of immune cells from Peyer's patches (PPs) and lamina propria (LP): PPs were dissected from the small intestine, pierced with a fine forcep. Tissues were digested in fresh RPMI-1640 medium containing 100 g/mL Liberase TM and 50ug/mL DNase I at 37C on a roto-mixer for 15 min. The medium was collected and added to ice-cold MACS buffer (pH 7.4; PBS with 2% FBS and 2mM EDTA). The remaining tissues were digested for another 15 min. Supernatants from the two steps were combined and passed through 70um strainer for further use.
To isolate cells from LP, the intestines were opened longitudinally and washed in ice-cold PBS. Then cut the intestines into 0.5-1.0 cm fragments after remove fat and PPs. Epithelial cells and intraepithelial lymphocytes (IELs) were dissociated by rotation (350rpm) in pre-warmed (37C) PBS containing 5% FBS, 10mM EDTA and 1mM DTT at 37C for 30 min. After washing with PBS containing 5% FBS, tissues were digested in pre-warmed (37C) RPMI-1640 medium with 10%FBS, 1mg/mL Collagenase IV (1.5 mg/mL for colon), 20 ug/mL DNase I, 10mM HEPES, 1mM Ca2+ and 1mM Mg2+ with 550rpm rotation for 20min at 37C. The supernatants were then passed through 70 m strainer after added to 2mM EDTA. Centrifuge at 4C, 500g for 5min. Then the pellets were resuspended in 3mL 40% Percoll, loaded on 1mL 80% Percoll, and centrifuged at 25C, 600g for 20min. Cells at the interphase of the two layers were collected and washed in MACS for counting, staining, culture and/or sorting.
Flow cytometry and cell sorting: After blocking nonspecific binding with Fc-blocking antibody 2.4G2 by incubation for 10 min on ice, single-cell suspensions were stained with surface-labeled antibodies (see Supplementary Table 1) for 20-30 min at 4C and then were washed with MACS buffer. In the experiment of detecting CD16 (Fcgr3), we used FBS instead of 2.4G2 for blocking.
For intracellular staining of transcription factor, the LPLs were fixed and permeabilized with Fixation/Permeabilization buffer (Foxp3 Transcription Factor Staining Buffer Kit, eBioscience) after surface markers staining. For intracellular staining of FcR1, BMDMs or LPLs were fixed and permeabilized with Fixation/Permeabilization Solution (BD) after surface markers staining, subsequently stained with anti- FcR1 antibody for 1 hour at 4C.
For analysis of phosphorylation of JAK1 and JAK3, the LPLs were stimulated with 100ng/mL rmIL-23 (R&D Systems) and rmIL-7 (Biolegend) in complete RPMI medium for 40min at 37C, 5% CO2. After stimulation, cells were stained with the surface-stained markers, subsequently fixed and permeabilized with pre-cooled methanol, then the cells were incubated for overnight at 4C with the indicated antibodies.
For intracellular staining of IL-22 and IL-17A, sorted CD45+live SSClow LPLs (Purity>92%) were stimulated with Cell Stimulation Cocktail, Protein Transport Inhibitor Cocktail (eBioscience) and 40ng/mL rmIL-23(R&D Systems) in complete RPMI medium (10% FBS, 10mM HEPES, 1mM sodium pyruvate, 80M 2-mercaptoethanol, 2mM glutamine, 100U/mL penicillin, 100 mg/mL streptomycin) for 2.5 or 4 hours when indicated at 37C, 5% CO2. After stimulation, cells were stained with the surface-stained markers, subsequently fixed and permeabilized with Fixation/Permeabilization Solution. After permeabilization, the cells were incubated for overnight at 4C with the indicated anti-cytokine antibodies.

Instrument

Flow cytometry was performed with CytoFLEX (Beckman Coulter) or LSR Fortessa (BD). For sorting, single-cell suspensions were sorted to > 97% purity after surface markers staining using a FACSAria Fusion (BD) or FACSAira III (BD) sorter with a 70um Nozzle at 4C.

Software

FACS data were collected by CytExpert software with CytoFLEX (Beckman Coulter), or BD FACSDiva™ Software with LSR Fortessa (BD), FACSAria Fusion (BD) or FACSAira III (BD). FACS data were analyzed by FlowJo software v10.0.7 (TreeStar).

Cell population abundance

The cell population abundances were shown on the flow plots. Post-sort purity was assessed by re-analyzing the sorted population and was always above 97% .

Gating strategy

Total live immune cells were gated according to CD45 and live/dead dye, then intact cells were gated according to the FSC-A and SSC-A. Doublets were excluded by FSC-H and FSC-A. Gating strategies for specific cell populations were illustrated in the supplementary figures.

☒ Tick this box to confirm that a figure exemplifying the gating strategy is provided in the Supplementary Information.
